# Zebularine showed anti-tumor efficacy in clear cell renal cell carcinoma

**DOI:** 10.3389/fphar.2025.1531056

**Published:** 2025-02-14

**Authors:** Haoyu Xu, Senlin Peng, Junwu Li, Yuanyuan Bai, Guozhi Zhao, Simin Liang, Wei Tang

**Affiliations:** Department of Urology, First Affiliated Hospital of Chongqing Medical University, Chongqing, China

**Keywords:** clear cell renal cell carcinoma, immune-related prognostic index, immunological profiling, drug screening, network pharmacology, molecular docking, Zebularine

## Abstract

**Background:**

Clear cell renal cell carcinoma (ccRCC) has the highest morbidity among renal cell carcinoma (RCC) subtypes. While existing clinical pharmacological intervention strategies have achieved certain efficacy, challenges including inevitable drug resistance and intricate immune heterogeneity of ccRCC continue to hinder their biomedical application. Therefore, developing novel immunotherapeutic agents and identifying patients who can gain the greatest benefits from these therapies are urgent issues.

**Methods:**

To address these challenges, mRNA expression profile and clinical data of ccRCC were obtained from The Cancer Genome Atlas (TCGA) and Gene Expression Omnibus (GEO) databases. These data were integrated and randomly allocated into training and test sets. Immune-related differentially expressed genes (IRDEGs) were used to construct an immune-related gene prognostic index (IRGPI). Both prognostic performance metrics and immune phenotyping were employed to evaluate the effectiveness of the model. Furthermore, model IRDEGs (mIRDEGs) in two risk subgroups were leveraged to select potential therapeutic compounds. Afterwards, network pharmacology and molecular docking techniques were used to elucidate the anti-cancer mechanisms of Zebularine (Zeb). Finally, the anti-cancer efficacy of Zeb was validated through *in vivo* and *in vitro* experiments.

**Results:**

Our constructed IRGPI exhibited superior prognostic performance. The drug screening revealed Zeb potentially targets the PI3K-Akt signaling pathway to exert its anti-cancer effects. Subsequent experimental validation corroborated these theoretical findings.

**Conclusion:**

This study presents a prognostic model to evaluate immune cell infiltration and predict the prognosis of ccRCC patients. The identified small molecule compound provides a novel therapeutic avenue for treating ccRCC patients.

## 1 Introduction

Clear cell renal cell carcinoma (ccRCC) constitutes approximately 70%–80% of renal cell carcinoma (RCC) and has the highest morbidity among kidney malignant neoplasms. It has a poor prognosis in advanced stages and is insensitive to conventional radiotherapy and chemotherapy (Li M. et al., 2023; Choueiri et al., 2020; Li T. et al., 2023). Although recent advancements in targeted therapies and innovative immunotherapies have significantly transformed the treatment paradigm for advanced ccRCC patients, many patients are unresponsive to therapies (Li et al., 2022; Choueiri et al., 2017), underscoring the necessity for further investigation into the biological characteristics and potential therapeutic biomarkers for ccRCC.

Existing studies have increasingly suggested that ccRCC is a heterogeneous disease, and its prognosis is not only influenced by clinical and pathological factors, but also closely related to immune status within tumor microenvironment (TME) (Lee et al., 2019; Bi et al., 2021). The activation and suppression states of the immune system directly impact patients’ response to immunotherapy (Kumari and Choi, 2022). As a result, developing an immune model that can accurately predict patient prognosis and identifying effective small molecule compounds are vital for formulating precise and individualized treatment strategies for ccRCC patients.

The development of prognostic models relies on comprehensively understanding patient biological features and identifying genes associated with tumor progression (Gogas et al., 2009). The prognosis of malignant tumors is intricately linked to immune responses, which encompass processes such as antigen presentation, phagocytosis, and lymphocyte activation (Phillips et al., 2019). To date, several immune-related prognostic models for ccRCC have been established. For instance, one study reveals novel features derived from macrophage marker genes through single-cell transcriptome data analysis, and identifies *IFI30*, *FUCA1*, *TIMP1*, *NAT8*, and *SMIM24* as the potential prognostic biomarkers (Chen et al., 2024). Moreover, investigations into lactate-related genes demonstrate that models constructed based on these genes significantly contribute to evaluating the prognosis and immune response in ccRCC (Sun et al., 2022). These multifactorial prognostic models, which incorporate various risk-related genes, can accurately predict the patient overall survival (OS) and progression-free survival (PFS).

Screening small molecule compounds can offer promising avenues for the treatment of ccRCC. Small molecule inhibitors targeting immune checkpoints (ICIs), such as *LAG-3* inhibitors (specifically SA-15-P), identified through focused screening and the “SAR by catalog” approach, can block *LAG-3/MHCII* and *LAG-3*/*FGL1* interactions. This mechanism assists T cells in regaining cytotoxicity while diminishing the immunosuppressive effect of regulatory T cells (Tregs), thereby highlighting their potential for clinical translation in immunotherapy for multi-organ solid tumors (Abdel-Rahman et al., 2023). Comparable investigations also encompass STING agonists identified via high-throughput screening, including Benzothiazine-6-carboxamide (Sali et al., 2015), which holds promising prospects for immunotherapeutic applications.

Network pharmacology integrates systems biology, network science and bioinformatics analysis to analyze drug-target molecular interactions at a systemic level. This approach systematically elucidates the drug action mechanisms, identifies novel drug targets, and optimizes drug design (Liu et al., 2021). Molecular docking technology is the pivotal tool used in computer-aided drug design (CADD), which is critical for forecasting interaction patterns and affinities between drug molecules and their targets (Bell and Zhang, 2019). Collectively, these methodologies establish the fundamental framework for applying small molecule compounds in anti-cancer therapy.

**FIGURE 1:**
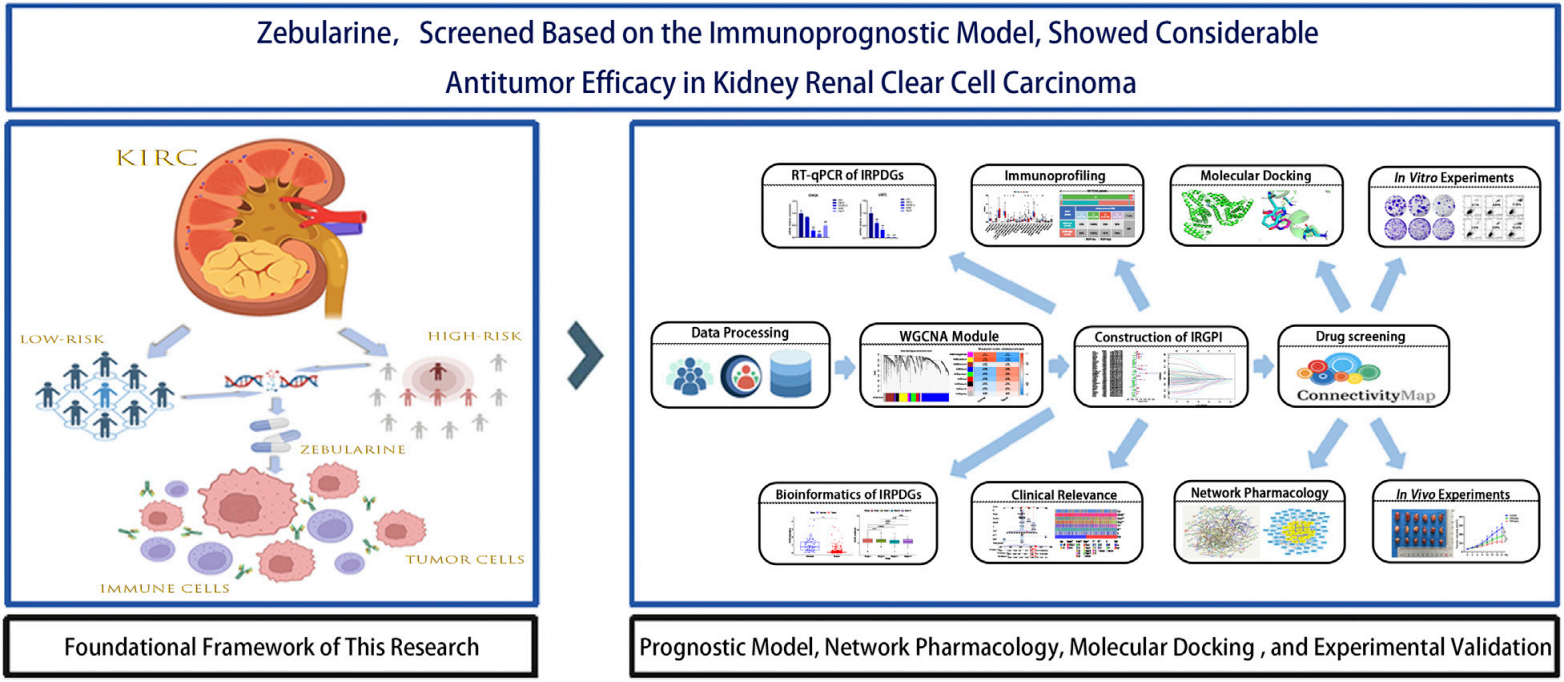
Flowchart of data collection and study design.

Therefore, based on this rationale, in this study, we used ccRCC data from The Cancer Genome Atlas (TCGA) and Gene Expression Omnibus (GEO) datasets to construct an 8-gene immune prognostic model for assessing ccRCC prognosis. Additionally, Connectivity map (CMap) database-based drug screening was conducted in conjunction with network pharmacology as well as *in vivo* and *in vitro* experimental validation to elucidate the anti-cancer efficacy of a novel small molecule compound. Figure 1 illustrates the key aspects and workflow of this study.

## 2 Methods

### 2.1 Raw datasets acquisition

The gene expression profiles and clinical data of ccRCC were downloaded from the TCGA database (https://portal.gdc.cancer.gov/, July 2024) (Stephen et al., 2014), which consist of 72 normal samples and 542 tumor samples. After excluding duplicate samples, we adopted a total of 537 ccRCC samples. Among them, 70% were randomized as the model training set, while the remaining 30% were combined with the GSE29609 dataset (consisting of 39 tumor samples with recorded survival times) from the GEO database (https://www.ncbi.nlm.nih.gov/geo/, July 2024) (Edgar and Barrett, 2006) as the test set to validate the model predictive ability. Following quality control, all datasets were standardized to ensure that the expression values were converted to comparable levels for further analysis. The detailed baseline clinical pathological data of patients with ccRCC are summarized in Table 1. Totally 3179 immune-related genes (IRGs) were obtained, including 1793 in ImmPort (https://www.immport.org/) (Bhattacharya et al., 2018) and 1698 in InnateDB (https://www.innateDB.com/) (Lynn et al., 2008) databases.

**TABLE 1:** Clinicopathological Characteristics of ccRCC Patients Examined in This Study.

Variables	Training set	Test set
Age (years)		
≤65	254	119
>65	122	81
Gender		
Male	242	107
Female	134	54
Unknow		39
Tissue		
Normal	72	
Tumor	376	200
Grade		
1	9	6
2	158	86
3	159	59
4	47	44
unknow	3	5
Stage		
I	190	91
II	39	21
III	89	46
IV	55	42
unknow	3	

### 2.2 Immune-related core module genes identification

For identifying differentially expressed genes (DEGs) in ccRCC tumor *versus* non-carcinoma samples, “limma” package of R software 4.4.1 was employed for screening DEGs from a training set comprising 376 ccRCC samples and 72 non-carcinoma samples upon the thresholds of P < 0.05 and |log2FC| > 1. Subsequently, these DEGs were intersected with IRGs downloaded to obtain immune-related differentially expressed genes (IRDEGs). Thereafter, key module genes were extracted from the IRDEGs by weighted gene co-expression network analysis (WGCNA) (Xu et al., 2022). Specifically, through Pearson test, sample cluster analysis was conducted according to the correlation coefficients between two genes. Later, an unsigned co-expression network was constructed in line with optimum soft-thresholding power β. The co-expression similarity was calculated, later, the similarity matrix was transformed to the weighted adjacency matrix. Afterwards, this adjacency matrix was further transformed to the Topological Overlap Matrix (TOM) for detecting network gene connectivity. Furthermore, genes with a distance of 1-TOM were clustered to construct different visual gene modules, and finally, genes from the significantly correlated modules were chosen in subsequent analyses.

### 2.3 Development and verification of the immune-related gene prognostic index (IRGPI)

To identify the OS-related independent and significant prognostic genes, univariate Cox (uni-COX) regression was performed. To mitigate model complexity and multicollinearity, Least Absolute Shrinkage and Selection Operator (LASSO) regression (McEligot et al., 2020) was carried out with R package “glmnet” and multivariate Cox (multi-Cox) regression for constructing an optimal immune-related prognostic differential genes (IRPDGs) model. Afterwards, risk score (RS) was determined through linearly combining gene expression and corresponding weighted regression coefficients obtained from multi-Cox regression. To be specific, the RS was computed below:

$$RS=\sum_{n=1}^{8}\text{Expression}\left({\text{gene}}_{n}\right)\times \text{coefficient}\left({\text{gene}}_{n}\right)$$

All samples were classified as high- or low-risk group according to median RS. At last, Kaplan-Meier survival and risk assessment were conducted on both training and test sets. Additionally, receiver operating characteristic (ROC) curves were plotted with “timeROC” package, with area under the curve (AUC) values being calculated to comprehensively assess and validate the predictive capability of IRGPI (Bilar et al., 2021).

In the training set, Chi-square test was employed for examining the relationship of prognosis with clinicopathological parameters (including age, gender, tumor stage, and grade). Additionally, RS and clinicopathological parameters were used for nomogram construction using “regplot” and “rms” packages. Thereafter, uni-Cox and multi-Cox regression were completed for assessing the independent predictive capability of IRGPI.

### 2.4 Analysis of mutant gene landscapes and immune characteristics

Mutation data were downloaded from the TCGA database and divided as high- or low-risk subgroup. The “maftools” package was employed for analyzing somatic mutations of each sample. Tumor mutation burden (TMB), survival analysis, and waterfall plots were utilized for evaluating somatic mutation difference between the two risk subgroups (Jardim et al., 2021). Thereafter, the “org.Hs.eg.db” and “clusterProfiler” packages were employed based on the reference gene set (c2.cp.kegg.v7.4.symbols.gmt) for gene set enrichment analysis (GSEA) of both risk subgroups (Zohair et al., 2023).

To explore the difference in immune characteristics, we implemented CIBERSORT analysis (https://CIBERSORT.stanford) for assessing relative proportions of 22 immune-related cells in the two risk subgroups. In addition, “survival” and “survminer” packages were adopted for survival analysis associated with immune cells, while “GSVA” package was utilized for single sample GSEA (ssGSEA) for 29 immune-related functional indicator levels. Furthermore, immune subtype datasets downloaded from TCGA database were utilized to match samples in the risk subgroups, thus assessing different immune subtypes in both risk subgroups, while TME-related scores were derived using the “estimate” package (Li H. et al., 2023). At last, the prognostic relevance of risk subgroups to immunotherapy and immune evasion was investigated by examining the difference in microsatellite instability (MSI), tumor immune dysfunction and exclusion (TIDE) score (https://tide.dfci.harvard.edu/), T-cell exclusion effects, and T-cell dysfunction (Qin et al., 2023).

### 2.5 Drug sensitivity analysis and small molecule drugs screening

For analyzing the ccRCC sensitivity to the common anti-cancer drugs, we utilized “pRRophetic” package for exploring sensitivity difference in both risk groups (Shi et al., 2023). Next, based on IRGPI subgroup classification, “limma” package was adopted for identifying model IRDEGs (mIRDEGs) from 188 high-risk and 188 low-risk samples (P < 0.05, |log2FC| > 0.5). Subsequently, 101 downregulated and 80 upregulated genes were uploaded to CMap database (https://clue.io) to identify potential therapeutic compounds (Subramanian et al., 2017). The screening results included negative changes in gene expression profiles of multiple cell lines, which were caused by 31,128 small molecule compounds, and the compound with the lowest negative score was selected as the candidate drug for high-risk ccRCC patients.

### 2.6 Acquisition of drug and disease targets

Briefly, the unique predicted targets for Zebularine (Zeb) were obtained from The Comparative Toxicogenomics Database (CTD, http://ctdbase.org/), STITCH 5.0 (http://stitch.embl.de/), DrugBank 6.0 (https://go.drugbank.com/), PharmMapper (http://www.lilab-ecust.cn/pharmmapper/), and SwissTargetPrediction (http://www.swisstargetprediction.ch/) databases (Davis et al., 2023; Szklarczyk et al., 2016; Knox et al., 2024; Wang et al., 2017; Daina et al., 2019). Besides, the DrugBank (https://go.drugbank.com/), GeneCards (https://www.genecards.org/), OMIM (https://www.OMIM.org/), and PharmGkb (https://www.pharmgkb.org/) databases (Shalar et al., 2005; Filatova et al., 2023; Altman, 2007) were searched with the keyword “clear cell renal cell carcinoma” to identify disease-related targets. Then, UniProt ID was transformed into Gene Symbol using UniProt database (https://www.uniprot.org/uploadlists/) (Apeweiler et al., 2004), the drug and disease targets were intersected after duplicates were removed, and the Venn diagrams were visualized utilizing the “venn” package.

### 2.7 Construction of the protein-protein interaction (PPI) network for core hub genes and pathway enrichment analysis

To identify overlapping targets, The intersection of DEGs (P < 0.05, |log2FC| > 1)from the TCGA-KIRC cohort with the established drug targets and disease genes were determined, and the results were visualized with a Venn diagram. Then, these target genes were imported into the Search Tool for the Retrieval of Interacting Genes/Proteins (STRING) database (https://cn.string-db.org/), whereas Cytoscape 3.10.2 software (https://www.cytoscape.org/) was applied in constructing the PPI network diagram (Szklarczyk et al., 2023). By using the CytoNCA plugin, the betweenness centrality (BC), closeness centrality (CC), degree centrality (DC), eigenvector centrality (EC), local average connectivity (LAC), and network centrality (NC) were calculated. Only genes with scores exceeding the median value across each metric column through two iterations of this process were retained as the core hub genes. Ultimately, Gene Ontology (GO) as well as Kyoto Encyclopedia of Genes and Genomes (KEGG) analysis was carried out for visualizing corresponding results of the core hub genes.

### 2.8 Vina molecular docking

For assessing binding energies of Zeb to its core hub targets and elucidate its interaction profile, two-dimensional (2D) Zeb structure was retrieved based on PubChem database (https://pubchem.ncbi.nlm.nih.gov/) before optimization with Chem3D (https://www.3dchem.com/) to obtain a three-dimensional (3D) conformation. In addition, the receptor crystal structure was sourced based on RCSB Protein Data Bank (PDB, https://www.rcsb.org/), while PyMOL 3.0 software (https://www.pymol.org/) was utilized to remove water molecules and extraneous ligands out of this structure. Molecular docking studies were then conducted utilizing AutoDock Tools 1.5.6 (http://autodock.scripps.edu/) and AutoDock Vina 1.1.2 (https://vina.scripps.edu/), and docking energy results were visualized. Furthermore, the key ligand-receptor interactions, including hydrogen bonds, hydrophobic interactions, and π-π stacking, were characterized using Protein-ligand Interaction Profiler (PLIP) database (http://plip-tool.biotec.tu-dresden.de/plip-web/plip/index/), followed by visualization with PyMOL (Ruan et al., 2023).

### 2.9 Comprehensive multi-faceted validation of the IRPDGs

We conducted an analysis on the expression difference of IRPDGs between tumor tissues and adjacent non-tumor tissues, utilizing the mRNA expression profile and clinically relevant data from TCGA-KIRC, employing the 'limma' package in R software. Additionally, we generated box plots using the “ggplot2” and “ggpubr” packages. The correlation between IRPDGs and clinical pathological factors was assessed through the “limma” package in R software. Subsequently, we retrieved immunohistochemistry (IHC) results for IRPDGs in normal *versus* tumor tissues from the Human Protein Atlas (HPA, https://www.proteinatlas.org/). Finally, we investigated the expression of IRPDGs across various cell clusters within single-cell datasets GSE111360, GSE139555, GSE121636, GSE159115 and GSE171306 sourced from the TISCH database (https://tisch.comp-genomics.org/).

### 2.10 Cell culture

The ccRCC cell lines 786-O, OS-RC-2, A498, 769-P and the human normal renal tubular epithelial cell line HK2 were maintained in the complete medium (90% RPMI-1640, 10% fetal bovine serum (FBS), and 1% penicillin-streptomycin), and inoculated into culture dishes or flasks for incubation under 37°C with 5% CO₂, with medium change at 2–3-day intervals. After reaching around 80%–90% of confluency, cells were digested using 0.25% trypsin and passaged or harvested for further experiments as per the experimental requirements.

### 2.11 RNA extraction and real-time quantitative polymerase chain reaction (RT-qPCR)

Total RNA was extracted from ccRCC cell lines 786-O, OS-RC-2, A498, 769-P, and the normal cell line HK2 using TRIzol reagent (Takara, Shiga, Japan). Complementary DNA (cDNA) synthesis was performed with the ABScript Neo Master Mix for qPCR with gDNA Remover (Abclone, Wuhan, China). Following the manufacturer’s instructions, RT-qPCR was conducted utilizing the 2X Universal SYBR Green Fast qPCR Mix kit (Abclone) along with specific primers designed for model genes. The relative expression of RNA was analyzed using the LightCycler^®^480 real-time fluorescence quantitative PCR system (Roche, Basel, Switzerland). All primer sequences were obtained from Tsingke Biotechnology Co., Ltd. (Beijing, China), with GAPDH being an internal reference gene. Primer sequences are provided in Supplementary Table.

### 2.12 3-(4, 5-dimethyl-2-thizolyl)-2, 5 diphenyltetrazolium bromide (MTT) toxicity assay

In brief, 786-O and OS-RC-2 cells (3000/well) were uniformly inoculated into 96-well plates. For evaluating *in vitro* effects of Zeb (MedChemExpress, NJ, United States) on the viability of ccRCC cell lines, Zeb at varying concentrations (0, 2.5, 5, 10, 20, 40, 80, and 160 μM) was used to treat both cell lines for 24, 48, and 72 h. Afterwards, every well was introduced 10 μL MTT reagent (Beyotime, Shanghai, China), followed by plate incubation for additional 4 h to facilitate formation of deep purple Formazan crystals. Subsequently, to dissolve the deep purple crystals completely, every well was introduced 100 μL Formazan solubilization solution (Beyotime, Shanghai, China). After complete crystal dissolution, absorbance was measured with the microplate reader at 570 nm. Then, cell viability curves and half maximal inhibitory concentration (IC50) were analyzed to illustrate the impact of the drug on cell viability.

### 2.13 Colony formation assay

The 786-O and OS-RC-2 cells (1000/well) were inoculated into 6-well plates and exposed to Zeb treatment at varying concentrations (20, 50, and 100 μM) for 2 weeks, with medium change every 3 days. Thereafter, phosphate-buffered saline (PBS) was added to wash the colonies before 20 min of fixation using 4% paraformaldehyde (v/v) and additional 30 min of crystal violet (0.1%, w/v, Beyotime, Shanghai, China) staining. Photographs was taken and colony number (>50 cells/colony) was counted with ImageJ software (Wayne Rasband, Bethesda, MD, United States).

### 2.14 Cell apoptosis assay

To assess whether Zeb induced apoptosis of ccRCC cells, 786-O and OS-RC-2 cells (5 × 10^5^/well) were inoculated into 6-well plates for overnight incubation. Subsequently, Zeb at varying concentrations (20, 50, and 100 μM) was added for 24 h of cell treatment, followed by trypsin digestion to prepare single-cell suspensions. After washing twice with cold PBS, the cells were stained using Annexin V-FITC and propidium iodide (PI). Cell apoptosis was analyzed through Cytoflex flow cytometry (Beckman Coulter, Brea, CA, United States).

### 2.15 Scratch assay

On reverse side of each well in the 12-well plate, three horizontal lines were delineated as reference markers. Later, the logarithmic-phase 786-O and OS-RC-2 cells were cultivated within the 12-well plate until the cell confluency grew to approximately 80%–90%. Subsequently, three parallel scratches perpendicular to the reference markers were made with the 200 µL pipette tip. Cells were exposed to 24 h of Zeb treatment at 20, 50 and 100 µM. The inverted microscope (Nikon, Tokyo, Japan) was employed to capture images of the scratched areas at 0 h (baseline) and 24 h (post-treatment), and relative migration rates were analyzed utilizing ImageJ software (Wayne Rasband).

### 2.16 Transwell assays

To perform the Transwell assays, we inoculated 786-O and OS-RC-2 cells (5 × 10^4^/well, 200 µL) in the upper chambers (pore size: 8 μm; Biofil, Guangzhou, China) pre-coated or uncoated with Matrigel (Beyotime, Shanghai, China), using serum-free RPMI-1640 medium. To facilitate chemotaxis, 10% FBS was added into the lower chambers as a chemical attractant to draw cells through the membrane. At 24 h post-incubation, a wet cotton swab was used to gently remove non-invading cells on the upper surface. While invading cells on the bottom side were subjected to 20 min of 4% paraformaldehyde fixation and 30 min of crystal violet solution (0.1%, w/v) staining. Upright microscopic examination (Olympus, Tokyo, Japan) was then conducted to capture images, while ImageJ software (Wayne Rasband) was employed for quantifying invading cell number within every field.

### 2.17 Western blot

Phenylmethylsulfonyl fluoride (PMSF, Servicebio, Wuhan, China) was added to RIPA lysis buffer (Servicebio) at a ratio of 1:100 for the lysis of both cells and tumor tissues. Protein concentrations were determined using a standard quantification method. Subsequently, proteins were separated by sodium dodecyl sulfate polyacrylamide gel electrophoresis (SDS-PAGE) and transferred onto polyvinylidene fluoride (PVDF) membranes (Servicebio). Membranes were blocked with 5% non-fat milk in Tris-buffered saline with Tween 20 (TBST) for 1 h at room temperature. Primary antibodies against Akt (1:1000; Abmart, T55561S, Shanghai, China), phosphorylated-Akt (p-Akt; 1:1000; Abmart, TA0016S, Shanghai, China), PI3K (1:1000; Affinity, AF6242, Changzhou, China), phosphorylated-PI3K (p-PI3K; 1:1000; UpingBio, YP-Ab-17845, Hangzhou, China) and β-actin (1:5000; Protaintech, 81115-1-RR, Wuhan, China) were incubated overnight at 4°C according to the manufacturer’s instructions. Following this, membranes were incubated with horseradish peroxidase (HRP)-conjugated secondary antibodies (1:5000; Servicebio, GB23303) for 30 min at room temperature. Protein bands were visualized using a ChemiScope S6 chemiluminescence imager (Clinx, Shanghai, China). The intensity of the relevant bands was quantified using ImageJ software (Wayne Rasband).

### 2.18 Xenograft models

The six-week-old female BALB/c nude mice were obtained from Changzhou Cavensis Experimental Animal Co., Ltd. (Changzhou, Jiangsu, China). All procedures involving animals were approved by the Institutional Animal Care and Use Committee of Chongqing Medical University (IACUC-CQMU), and approval number is IACUC-CQMU-2024-0664. The OS-RC-2 cell line xenograft model was employed to assess the efficacy of Zeb in inhibiting ccRCC growth. Briefly, OS-RC-2 cells were subcutaneously injected into the right dorsal flank of BALB/c nude mice at a concentration of 2.5 × 10^6^ cells per 100 µL PBS. Once tumor volumes reached 50 mm³, the mice were stratified into three groups, each consisting of six animals. Mice in the experimental groups received oral administration of Zeb at doses of 250 mg/kg and 500 mg/kg every other day, while those in the control group were treated with PBS. Upon reaching predetermined tumor sizes, mice were euthanized; tumors were excised, photographed, measured, weighed, and recorded. The tumor volume (V) was calculated using the following formula: V (mm3) = 0.5 × length × width × width. The heart, liver, lung, spleen, kidney tissues along with tumors were fixed in 4% paraformaldehyde and subsequently embedded in paraffin for further analysis including *hematoxylin-eosin* (H&E) staining (ZSGB-BIO, Beijing, China), Ki67 immunohistochemistry (HUABIO, Hangzhou, China) and TUNEL fluorescence assay (Beyotime, Shanghai, China). Finally, we conducted a toxicity evaluation of Zeb in relation to commonly used first-line agents for metastatic ccRCC utilizing the ProTox database (https://tox.charite.de/).

### 2.19 Statistical analysis

This study completed statistical analysis with GraphPad Prism 10.3 (Inc., CA, United States). Data were represented as mean ± standard deviation (SD). Among-group differences were compared by one-way or two-way analysis of variance (ANOVA). P < 0.05 stood for statistical significance.

## 3 Results

### 3.1 Core immune-related gene identification

**FIGURE 2:**
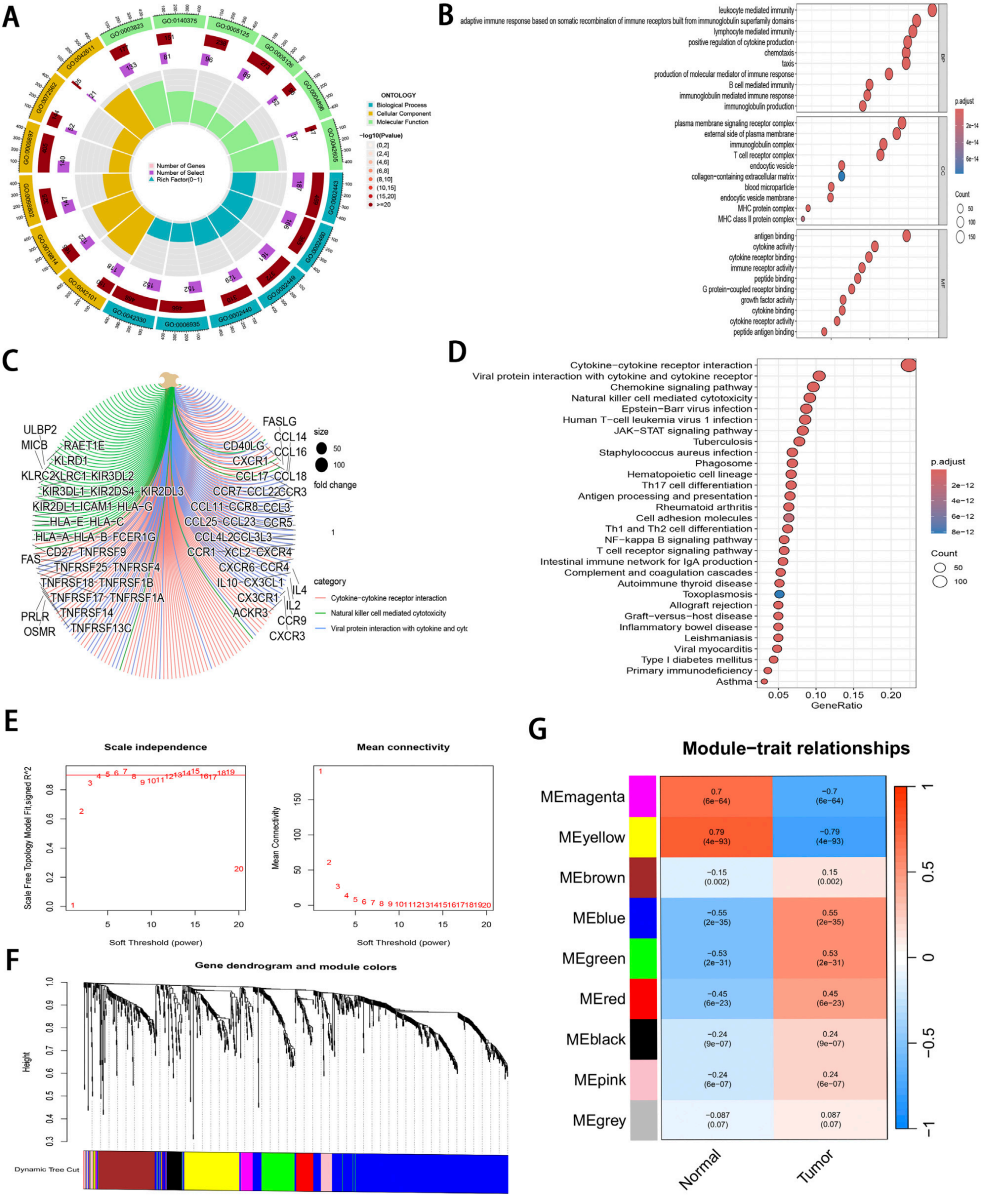
Pathway enrichment analysis and WGCNA of IRDEGs. **(A)** A circle diagram of GO annotation. **(B)** Top 10 terms in BP, CC, and MF categories of GO annotation of IRDEGs in training set. **(C)** A gene-pathway interaction network diagram based on KEGG analysis. **(D)** Top 30 pathways identified by KEGG analysis of IRDEGs in training set. **(E)** Scale independence and mean connectivity metrics for IRDEGs in training set. **(F)** A dendrogram illustrating gene clustering for IRDEGs in training set. **(G)** Merged modules and correlation analyses obtained from WGCNA for IRDEGs in training set.

DEGs related to ccRCC were extracted from the training set and intersected with IRGs identified by ImmPort and InnateDB, as a result, altogether 1,065 IRDEGs were identified (Supplementary Figure S1A). Upon GO annotation, the circular diagram is presented in Figures 2A, 10 most significantly enriched pathways in biological processes (BP), cellular components (CC), and molecular functions (MF) are delineated in Figure 2B (P < 0.001). Meanwhile, the gene-pathway interaction network (Figure 2C) and 30 most significantly enriched pathways were acquired from KEGG analysis (P < 0.001; Figure 2D). Subsequently, WGCNA was employed to cluster the IRGEGs based on sample data. By using a scale-free topology model, an optimal soft threshold of 3 was determined for this study, corresponding to a correlation coefficient of 0.9 (Figure 2E). Genes were later clustered according to their distance matrix, allowing for dynamic identification of neighboring genes and module formation. Modules with high similarity were merged, therefore, IRDEGs were assigned into nine distinct modules, as shown in Figures 2F, G. Ultimately, the yellow module that exhibited the most significant association with tumor status was identified based on P-value (P = 4e^−93^), and altogether 142 core immune-related genes were obtained for further investigation.

### 3.2 Development and validation of the IRGPI and analysis of its clinical relevance

**FIGURE 3:**
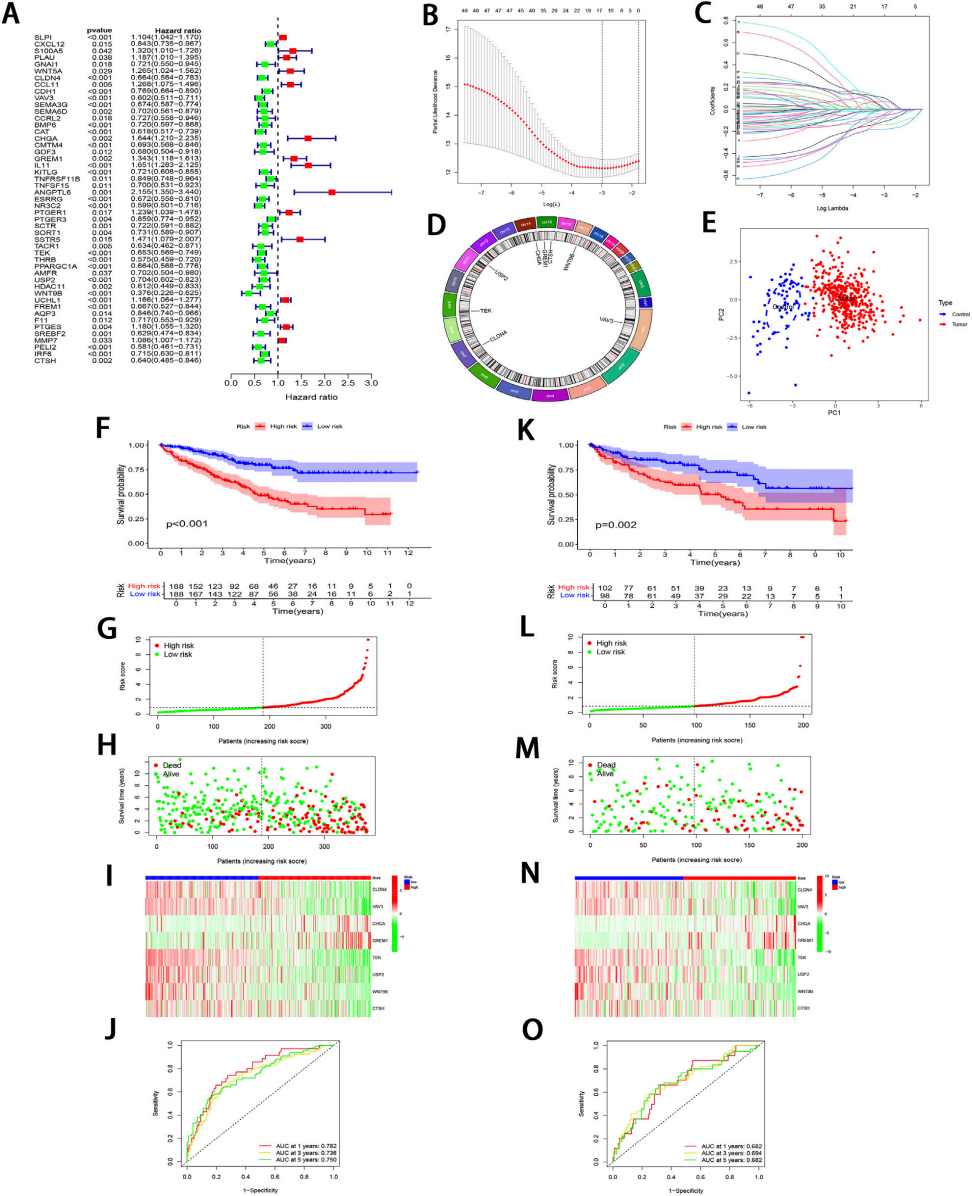
Development and validation of IRGPI. **(A)** Forest plot illustrating the uni-Cox regression analysis of 49 IRDEGs based on OS. **(B)** A profile plot showing the coefficients for the logarithmic (λ) sequence. **(C)** LASSO coefficient profiles for candidate genes among IRDEGs. **(D)** A chromosomal localization map of the eight IRPDGs. **(E)** PCA distribution plot in the normal and tumor groups. **(F)** Comparative analysis of survival curves between two patient subgroups in training set. **(G, H)** RS and survival status distribution in all samples of training set based on IRGPI. **(I)** Heatmap showing 8 IRPDGs expression among all samples in training set according to IRGPI. **(J)** ROC curves representing 1-, 3-, and 5-year OS in training set. **(K–O)** Model accuracy validated in the test set using methods analogous to those described in **(F–J)**.

Through uni-Cox regression analysis, 49 IRGs significantly related to ccRCC prognosis were obtained and illustrated using a forest plot (P < 0.05; Figure 3A). Subsequently, LASSO regression (Figures 3B, C) combined with multi-Cox regression confirmed 8 IRPDGs (*CLDN4*, *VAV3*, *CHGA*, *GREM1*, *TEK*, *USP2*, *WNT9B*, and *CTSH*) as the core genes of the IRGPI (P < 0.05). The survival analyses of each core gene within the model are presented in Supplementary Figure S1B (P < 0.001), while those for the remaining 41 prognosis-related genes are illustrated in Supplementary Figure S2 (P < 0.01). Figure 3D illustrates the chromosome circle maps of the eight IRPDGs, The Principal Component Analysis (PCA) distribution plot demonstrates that the eight-gene model effectively distinguishes the normal and tumor groups (Figure 3E).The RS was calculated as follows: *CLDN4* expression × (−0.204339644182801) + *VAV3* expression × (−0.208148784601721) + *CHGA* expression × (0.293414690410473) + *GREM1* expression × (0.27431516170263) + *TEK* expression × (−0.268132296798697) + *USP2* expression × (−0.12985305134718) + *WNT9B* expression × (−0.3531340919342) + *CTSH* expression × (−0.202002927826549). Samples were stratified as high- or low-risk subgroup according to the median RS, The low-risk subgroup showed significantly superior prognosis to high-risk subgroup (P < 0.01; Figures 3F–I). Moreover, as indicated by ROC curves, 1-, 3- and 5-year AUCs were 0.782, 0.736, and 0.750, separately (Figure 3J), demonstrating that the constructed IRGPI model exhibited strong predictive performance. Furthermore, the results from the test set used for validation were consistent with those obtained from the training set (Figures 3K–O).

**FIGURE 4:**
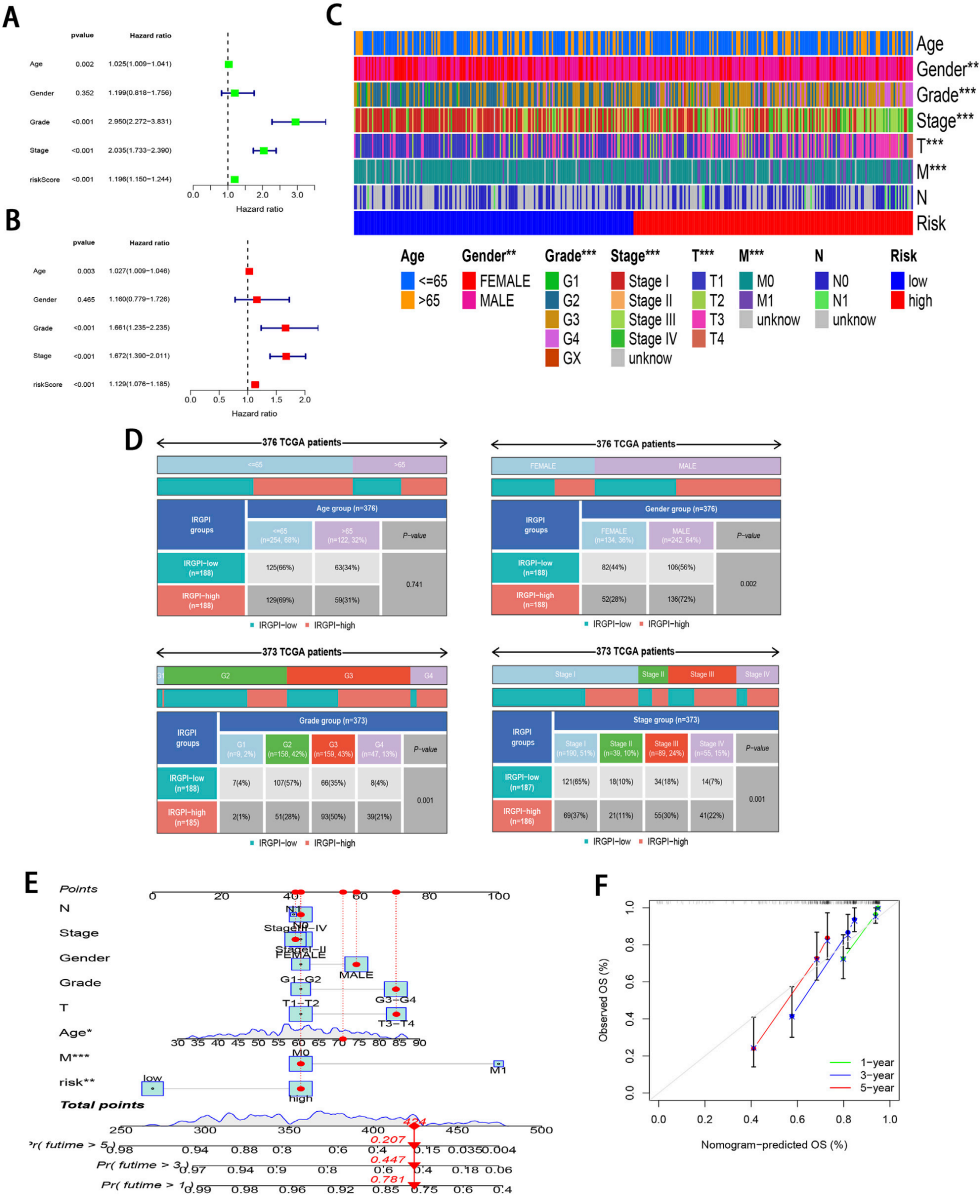
Clinical relevance analysis of subgroups. **(A, B)** Uni-Cox and multi-Cox regression exploring relationship of clinicopathological factors and RS with OS. **(C, D)** Heatmap and tabulated representation of the distribution of clinicopathological factors across subgroups. **(E)** Nomograms for predicting 1-, 3-, and 5-year survival according to clinicopathological factors and RS. **(F)** Calibration curves comparing the observed OS with predicted survivals at 1, 3, and 5 years.

To further validate the significant role of IRGPI in ccRCC, Cox regression was carried out for exploring the relationships of prognosis with clinicopathological parameters. Uni-Cox regression identified age, grade, stage, and RS as the high-risk factors for ccRCC prognosis (P < 0.01; Figure 4A). Additionally, multi-Cox regression analysis demonstrated that, compared with other variables, these parameters functioned as the independent prognostic factors (P < 0.01; Figure 4B). From Chi-square tests for clinical correlation analysis, there were significant difference in gender, grade, and stage (including T and M stages) between the two subgroups (P < 0.01; Figures 4C, D). By integrating all parameters, a nomogram that exhibited excellent concordance with the observed OS was developed (Figures 4E, F).

### 3.3 GSEA and TMB

**FIGURE 5:**
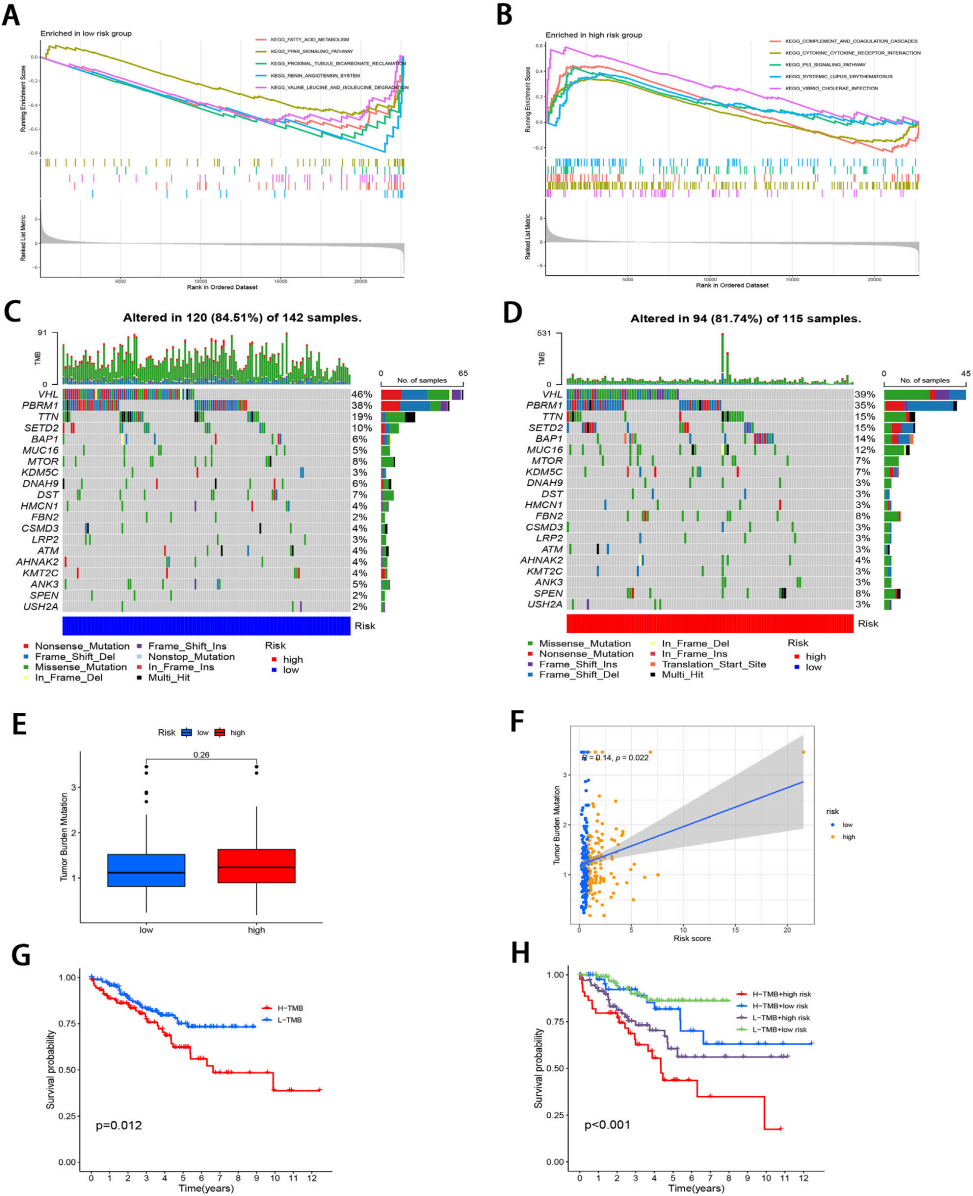
Molecular characterization of subgroups. **(A, B)** GSEA of two subgroups. **(C, D)** Mutation landscape plots depicting 20 most highly mutated genes in two subgroups. **(E)** Comparative analysis of difference in TMB of both subgroups. **(F)** Correlation distribution between RS and TMB. **(G)** Survival curves comparing the high- and low-TMB groups. **(H)** Stratified survival curves for the combinations of RS and TMB.

To investigate the pathways in the two risk groups, GSEA was carried out. It was found that fatty acid metabolism, PPAR signaling pathway, proximal tubule bicarbonate reclamation, renin angiotensin system, and the degradation of specific amino acids were significantly enriched in the low-risk group (P < 0.001; Figure 5A). On the contrary, pathways associated with complement and coagulation cascades, cytokine receptor interactions, P53 signaling pathway, systemic lupus erythematosus, and *vibrio cholerae* infection were markedly enriched in the high-risk group (P < 0.05; Figure 5B).

Subsequently, “maftools” package was utilized to analyze the distribution patterns of those 20 most frequent somatic mutation genes between the two subgroups, which revealed distinct landscape variations (Figures 5C, D). Notably, *VHL*, *PBRM1*, *TTN*, *SETD2*, and *BAP1* emerged as the most frequently mutated genes. TMB, defined as the number of mutated bases per million bases in tumor tissue samples, has surfaced as the candidate prognostic biomarker (Singal et al., 2019). As illustrated in Figure 5E, the TMB level elevated in high-risk subgroup relative to low-risk subgroup, with no significant difference (P = 0.26). However, TMB level was significantly and positively related to RS (r = 0.14; P < 0.05; Figure 5F). To delve deeper into the synergistic prognostic implications of TMB and RS, it was found that the lower TMB level was related to the higher survival probability (P < 0.05; Figure 5G). Moreover, the combination of TMB level and RS demonstrated superior stratified prognosis prediction capability for ccRCC patients (P < 0.001; Figure 5H).

### 3.4 Immunological profiling

**FIGURE 6:**
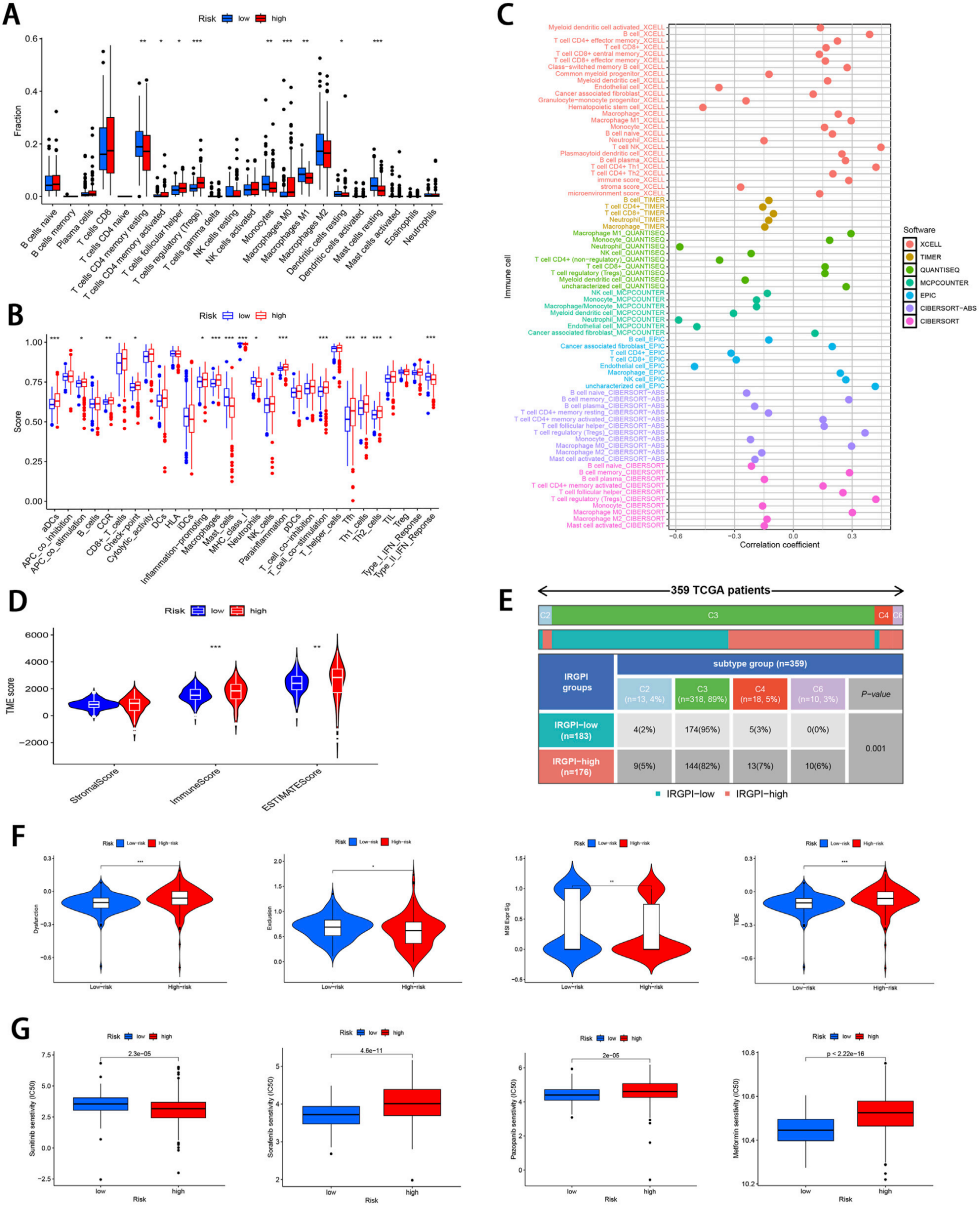
Comprehensive immune cell profiling of different subgroups. **(A)** Distribution of 22 immune cell types between the two subgroups. **(B)** Variations in the distribution of 29 immune-related functions between the two subgroups. **(C)** Correlation distribution between RS and various immune cell types as calculated by different algorithms. **(D)** Comparative analysis of TME-related indicator scores between the two subgroups. **(E)** Tabulated distribution of immune subtypes (C2, C3, C4, and C6) in the subgroups. **(F)** Difference in T-cell dysfunction, exclusion, MSI, and TIDE scores between the two subgroups. **(G)** Difference in the sensitivity to primary first-line therapies for ccRCC and Metformin between the two subgroups (*p < 0.05, **p < 0.01, ***p < 0.001).

The 22 immune cell proportions were quantified across all samples using CIBERSORT (Figure 6A). It was found that resting CD4 memory T cells, monocytes, M1 macrophages, resting mast cells, and resting dendritic cells were significantly infiltrated in the low-risk group. Conversely, follicular helper T cells, activated CD4 memory T cells, M0 macrophages and regulatory T cells were significantly infiltrated in the high-risk group (P < 0.05), suggesting that ccRCC progression was closely related to these cell types. Furthermore, nearly all factors of significant immune-related functions were markedly expressed in the high-risk group, such as antigen-presenting cell (APC) co-stimulation, activated dendritic cells (aDCs), checkpoint pathways, cytolytic activity, chemokine receptor signaling (CCR), inflammation promotion mechanisms, macrophage activity, para-inflammation processes, T cell co-stimulation dynamics as well as follicular helper T cells, T helper cell 1 (TH1), and T helper cell 2 (TH2) responses alongside tumor-infiltrating lymphocytes (TIL) (P < 0.05; Figure 6B). Survival analysis pertaining to 10 immune cell types is illustrated in Supplementary Figure S3 (P < 0.05). Figure 6C; Supplementary Figure S4 illustrate the relative distribution of various immune cell types with RS across different algorithms (P < 0.05).

Subsequently, the “estimate” package was employed to calculate stromal/immune/estimate scores within TME of both subgroups. As illustrated in Figure 6D, individuals in the high-risk group showed markedly higher immune scores and estimate scores (P < 0.01), revealing a reduced proportion of tumor cells, and the increased immune cell and immune-related molecule diversity. Previous studies have identified 6 kinds of distinct infiltrating immune cell subtypes in cancer patients that may either promote or inhibit tumor cell proliferation (Tamborero et al., 2018). In this investigation, the C3 subtype (Inflammatory) was predominant in the low-risk group, whereas C2 (IFN-γ dominant), C4 (lymphocyte depleted), and C6 (TGF-β dominant) subtypes were predominantly observed in the high-risk group (P < 0.001; Figure 6E). Moreover, it was observed that the C3 subtype was correlated with favorable prognosis, while C4 and C6 subtypes were linked to poor outcomes (Vesteinn et al., 2018), underscoring the validity of immune subtyping based on the IRGPI.

Finally, the TIDE score was adopted to assess the potential for immune escape. As illustrated in Figure 6F, the high-risk group showed elevated TIDE and Dysfunction scores, signifying the greater propensity for immune evasion and reduced sensitivity to ICIs (P < 0.001). For the low-risk group, MSI and T cell exclusion were the predominant factors (P < 0.05).

### 3.5 Comparison of drug sensitivity and identifying small molecule compounds

To evaluate the efficacy of targeted therapies in the two risk subgroups and to identify new therapeutic agents for ccRCC, the sensitivity of first-line therapies for metastatic ccRCC was analyzed between the two subgroups utilizing the “pRRophetic” package. As illustrated in Figure 6G, Sorafenib (P = 4.6e^−11^) and Pazopanib (P = 2e^−5^) demonstrated superior treatment sensitivity in the low-risk subgroup, whereas Sunitinib exhibited a contrasting effect (P = 2.3e^−5^). Notably, the hypoglycemic agent Metformin seemed to show an enhanced efficacy in low-risk patients compared with Sorafinib and Pazopanib (P = 2.22e^−16^). The potential small molecule compounds that might be beneficial for high-risk individuals were screened by analyzing mIRDEGs (P < 0.05; |log2FC| > 0.5; Supplementary Figures S5A, B) using the CMap platform. The top 10 therapeutic agents are summarized in Table 2. Zeb, with the lowest score among the evaluated therapeutic agents was identified. Zeb is a DNA methyltransferase inhibitor that can induce cell cycle arrest and apoptosis through intrinsic apoptotic pathway by activating *BAX* and *BAK* (Ruiz-Magana et al., 2012). There are currently no reports addressing whether Zeb influences the progression of ccRCC. Therefore, the role of Zeb was investigated in the following experiments.

**TABLE 2:** CMap platform small molecule compound screening.

Rank	Name	Raw connectivity score	Normalized connectivity score	FDR q nlog10
1	Zebularine	−0.7194	−1.836	15.6536
2	Thiotepa	−0.7179	−1.8321	15.6536
3	Guanethidine	−0.7064	−1.8028	15.6536
4	BRD-K87426959	−0.7022	−1.792	15.6536
5	BRD-K51971121	−0.7007	−1.7882	15.6536
6	BRD-K98824517	−0.7	−1.7864	15.6536
7	Danegaptide	−0.6999	−1.7863	15.6536
8	Formestane	−0.6964	−1.7772	15.6536
9	BRD-K95349679	−0.6935	−1.77	15.6536
10	Erythromycin-ethylsuccinate	−0.6913	−1.7644	15.6536

### 3.6 Prediction of core targets of Zeb in ccRCC and establishment of PPI network

**FIGURE 7:**
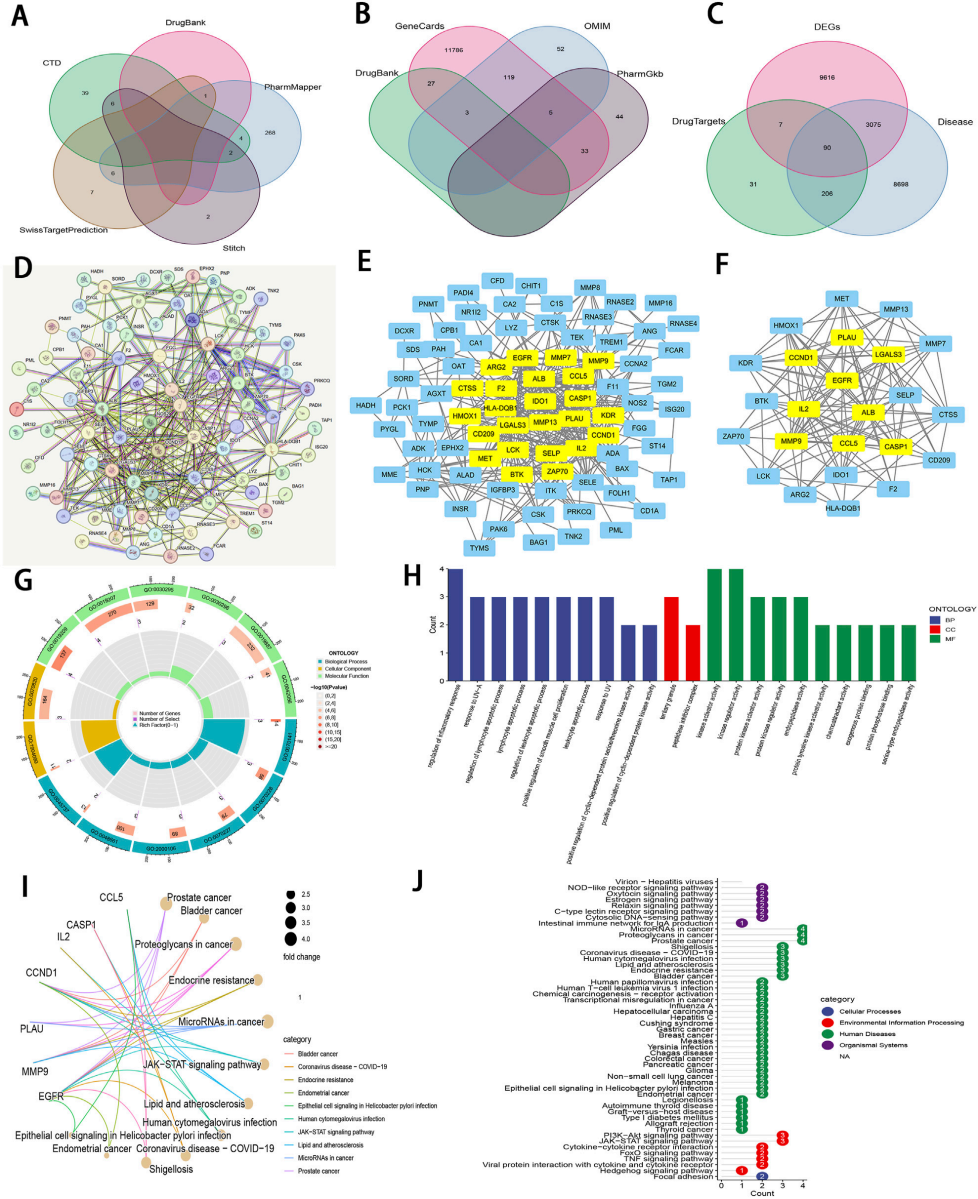
An integrative network pharmacology analysis of Zeb targeting ccRCC **(A)** The Venn diagram illustrating Zeb’s target based on different databases. **(B)** A Venn diagram depicting targets of ccRCC-related genes from multiple databases. **(C)** A Venn diagram illustrating the intersections between Zeb and ccRCC-related targets, as well as DEGs from TCGA-KIRC. **(D)** A PPI network generated by the STRING platform for the intersected targets of Zeb and ccRCC, with the medium confidence being set to >0.4 and free nodes being hidden. **(E)** The PPI network established based on Cytoscape software and interaction file for the intersected targets obtained from STRING platform. **(F)** A PPI network of core hub genes obtained after CytoNCA screening. **(G)** A circle diagram of GO annotation for core hub genes. **(H)** Top 10 terms in BP, CC, and MF categories from GO annotation of core hub genes. **(I)** A gene-pathway interaction network diagram of core hub genes from KEGG analysis. **(J)** A total of 51 distinct pathways obtained by KEGG enrichment of core hub genes.

Zeb’s targets were obtained from the CTD, STITCH 5.0, DrugBank, PharmMapper, and SwissTargetPrediction databases. Thereafter, these targets were converted from UniProt identifiers to standardized gene names and duplicates were removed, resulting in a total of 335 unique targets (Figure 7A). Additionally, 12,069 ccRCC-related targets were also identified based on DrugBank, GeneCards, OMIM, and PharmGkb databases (Figure 7B). By intersecting 12,788 DEGs derived from TCGA database of 537 tumor samples and 72 adjacent samples with both the 355 targets of Zeb and the 12,069 targets of ccRCC, a set of 90 common targets was obtained (Figure 7C).

These 90 shared targets were imported into STRING online platform (with *Homo sapiens* being the organism, medium confidence >0.4). After concealing the free-floating nodes (genes), the PPI network including 83 nodes and 706 edges (interactions) was acquired (Figure 7D), suggesting that Zeb might exert its influence on ccRCC through these genes. This PPI network file was uploaded in Cytoscape 3.10.2, where CytoNCA plugin was used to calculate BC, CC, DC, EC, LAC, and NC scores. High-score nodes were identified according to their scores exceeding the median value for each metric through two rounds of iterations, ultimately yielding 9 core targets, namely, *ALB* (albumin), *CASP1* (caspase-1), *CCL5* (C-C motif chemokine ligand 5), *CCND1* (cyclin D1), *EGFR* (epidermal growth Factor Receptor), *IL2* (interleukin-2), *LGAL3* (galectin-3), *MMP9* (matrix metalloprotease-9), and *PLAU* (plasminogen activator, urokinase) (Figures 7E, F).

### 3.7 GO and KEGG analysis for the core targets

Those 9 core genes identified were subjected to GO and KEGG analysis, leading to 497 GO terms and 51 KEGG pathways. Among them, the core targets mainly participated in the regulation of inflammatory response, response to UV−A, lymphocyte apoptotic process, regulation of lymphocyte apoptotic process, and regulation of leukocyte apoptotic process in BP. Regarding CC, these core targets were mainly related to tertiary granule and peptidase inhibitor complex. The MF terms enriched by these core targets included kinase regulator activity, kinase activator activity, protein kinase regulator activity, protein kinase activator activity, and endopeptidase activity (P < 0.01; Figures 7G, H).

The gene-pathway interaction network (Figure 7I) and 51 significant pathways were visualized through KEGG analysis (P < 0.01; Figure 7J). Within the 4 categories of KEGG, Organismal Systems encompassed pathways such as Virion-Hepatitis viruses, NOD-like receptor signaling pathway, Oxytocin signaling pathway. Human Diseases comprised MicroRNAs in cancer, proteoglycans in cancer, prostate cancer, bladder cancer, endocrine resistance, lipid and atherosclerosis, and more. PI3K-Akt signaling pathway, JAK-STAT signaling pathway, cytokine-cytokine receptor interaction, FoxO signaling pathway, TNF signaling pathway, viral protein interaction with cytokine and cytokine receptor, and hedgehog signaling pathway were classified under Environmental Information Processing, while Focal adhesion was categorized within Cellular Processes. Notably, PI3K−Akt, JAK−STAT, and TNF signaling pathways are important for ccRCC occurrence and development (Miao et al., 2018), (Liu et al., 2022). The corresponding schematic representations of the three pathways are illustrated in Supplementary Figure S5C.

### 3.8 Molecular docking

**FIGURE 8:**
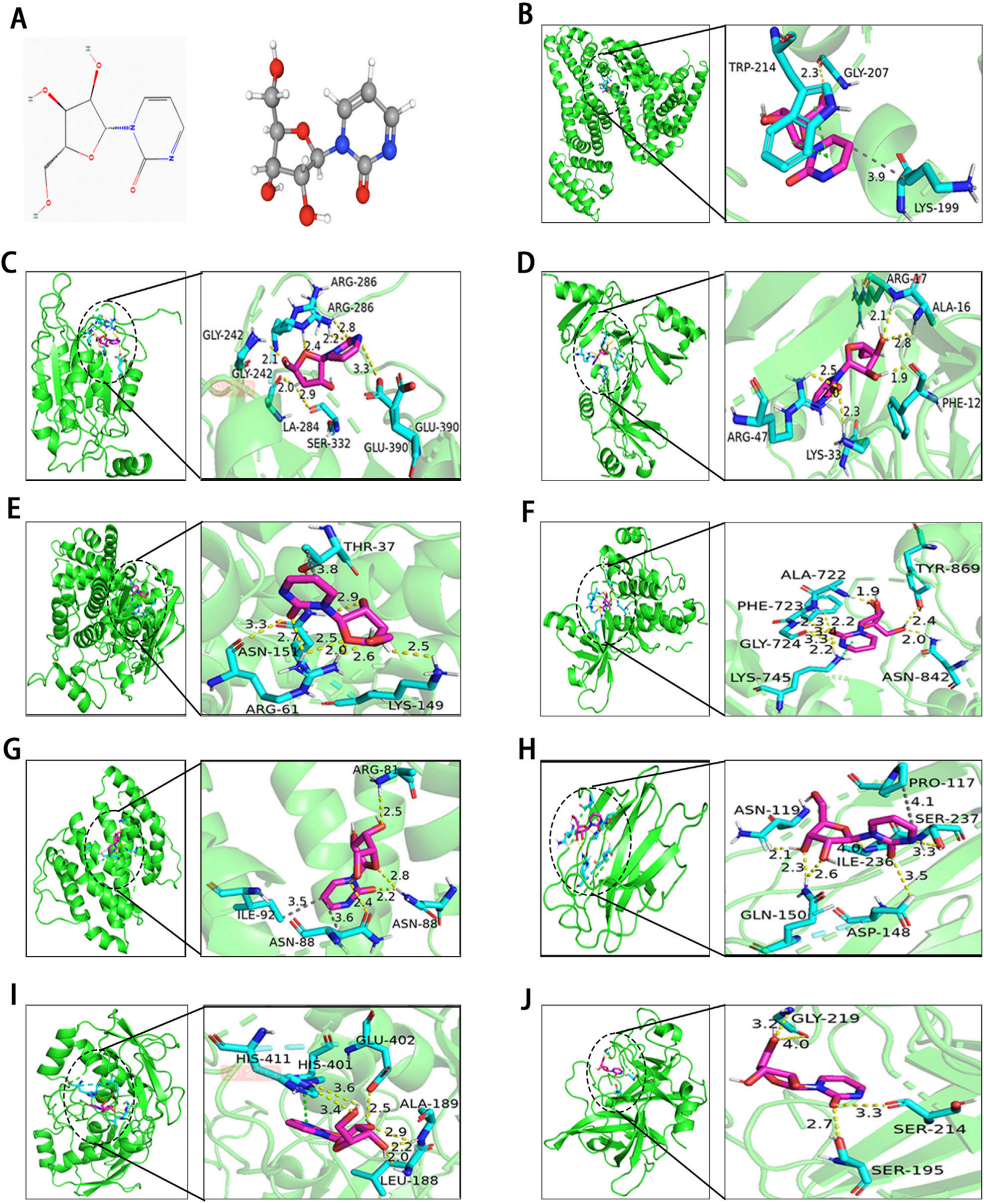
Results of molecular docking between receptors and ligands. **(A)** Visualization of Zeb’s 2D and 3D structures based on the PhbChem database. **(B–J)** Molecular docking results of Zeb targeting the core hub genes: **(B)** *ALB*, **(C)** *CASP1*, **(D)** *CCL5*, **(E)** *CCND1*, **(F)** *EGFR*, **(G)** *IL-2*, **(H)** *LGALS3*, **(I)** *MMP9*, and **(J)** *PLAU*. (Yellow, gray and green dashed lines indicate hydrogen bonds, hydrophobic interactions, and π-π stacking separately).

To evaluate the binding affinity of Zeb to its core targets, we conducted molecular docking studies. First, Figure 8A illustrated both the 2D and 3D structures of Zeb. Thereafter, the PDB IDs for the major receptors were retrieved from PDB database. AutoDock Tools was employed to define the active site pockets and grid dimensions. Subsequently, molecular docking simulations were performed using AutoDock Vina, with the resulting 3D interaction diagrams presented in Figures 8B–J. Detailed binding parameters are summarized in Table 3. The binding energies of all Zeb-receptor complexes were consistently below −5 kcal/mol, with an average binding energy of −6.5 kcal/mol across the nine receptor systems. These results indicate that Zeb exhibits robust and stable binding interactions with its core targets, highlighting the potential for downstream signaling pathway activation.

**TABLE 3:** Detailed binding information of Zeb with core hub targets.

Target gene	PDB ID	Active pocket coordinates	Grid dimensions	Binding energy (kcal/mol)
*ALB*	1GNI	X = 25.756, Y = 9.449, Z = 9.866	X = 40, Y = 40, Z = 40	−6.6
*CASP1*	8WRA	X = 14.119, Y = −1.773, Z = −4.46	X = 40, Y = 40, Z = 40	−8.4
*CCL5*	2VXW	X = 6.515, Y = −13.943, Z = −14.448	X = 40, Y = 40, Z = 40	−6.0
*CCND1*	2W96	X = 5.936, Y = 3.653, Z = 59.281	X = 40, Y = 40, Z = 40	−5.9
*EGFR*	8A2D	X = −15.9, Y = −7.27, Z = 16.34	X = 40, Y = 40, Z = 40	−6.7
*IL-2*	1M48	X = 11.81, Y = 1.491, Z = 13.909	X = 40, Y = 40, Z = 40	−5.9
*LGALS3*	3ZSJ	X = −10.421, Y = 0.34, Z = 5.536	X = 40, Y = 40, Z = 40	−6.1
*MMP9*	1GKC	X = 54.587, Y = 21.248, Z = 129.553	X = 40, Y = 40, Z = 40	−6.7
*PLAU*	5YC6	X = −28.328, Y = −17.453, Z = 0.419	X = 40, Y = 40, Z = 40	−6.6

Figure 8B illustrates the interaction of Zeb with *ALB* through various intermolecular forces, including hydrogen bonds with residue GLY-207, hydrophobic interactions with LYS-199, and π-π stacking with TRP214. In the binding of *CASP1* and *CCL5*, Zeb primarily interacts with the corresponding residues via hydrogen bonds (Figures 8C, D). Figure 8E highlights a single-residue multi-site binding pattern, particularly the interaction of Zeb with the ARG-61 residue of *CCND1*. The binding mode of Zeb-*EGFR* exemplifies single-residue and multiple-site, multi-residue and single-site, and diverse intermolecular forces (Figure 8F). Figure 8G demonstrates multiple intermolecular interactions on the same residue, notably the binding with ASN-88. Hydrogen bonds and hydrophobic interactions dominate the Zeb-*LGALS3* complex (Figure 8H), while hydrogen bonds and π-π stacking are predominant in the Zeb-*MMP9* interaction (Figure 8I). The binding of Zeb to *PLAU* is relatively simple, with GLY-219, SER-214, and SER-195 forming hydrogen bonds with Zeb (Figure 8J).

### 3.9 Characteristics and RT-qPCR analysis of IRPDGs

**FIGURE 9:**
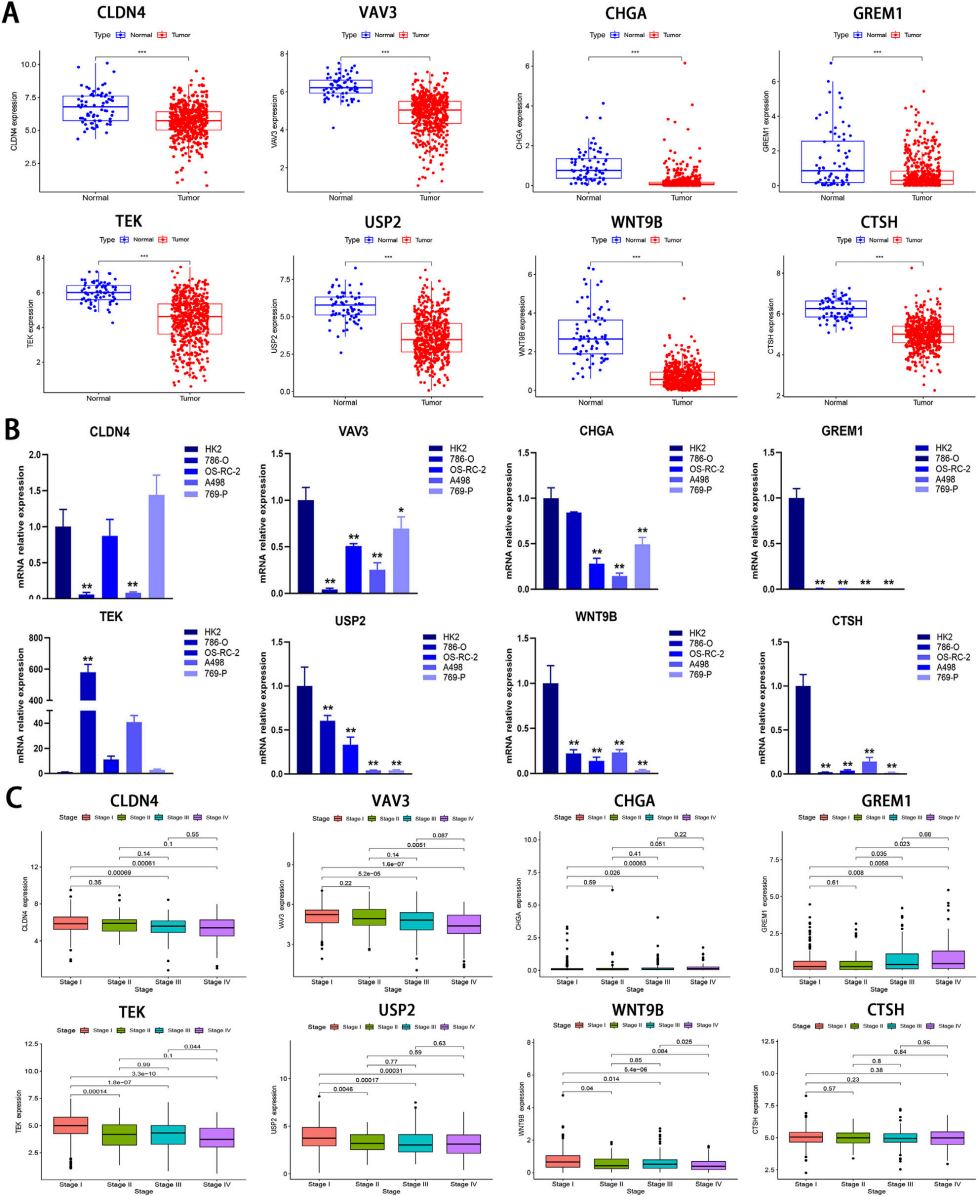
Bioinformatics and RT-qPCR analysis of IRPDGs. **(A)** Differential expression of eight model genes between the normal and tumor groups in the TCGA-KIRC cohort. **(B)** Relative expression levels of eight model genes assessed by RT-qPCR in 4 ccRCC cell lines and one normal cell line. **(C)** The association between eight model genes and clinical staging. (*p < 0.05, **p < 0.01, ***p < 0.001 *versus* Control; n = 3).

To investigate the differential expression of the eight model genes, we analyzed their expression profiles between tumor tissues and normal tissues using data from the TCGA-KIRC cohort. As illustrated in Figure 9A, all eight model genes exhibited downregulation in tumor tissues (P < 0.001). Subsequent RT-qPCR assays demonstrated that *VAV3*, *CHGA*, *GREM1*, *USP2*, *WNT9B*, and *CTSH* exhibited significantly lower expression levels in the four tumor cell lines than those in the HK2 cell line (P < 0.05; Figure 9B), corroborating the findings presented in Figure 9A. For *CLDN4*, significant downregulation was observed in both 786-O and A498 cell lines (P < 0.01); however, no significant difference were noted when compared OS-RC-2 and 769-P with HK2. In contrast, *TEK* showed upregulation across all tumor cell lines but only displayed statistical significance in the 786-O cell line (P < 0.01; Figure 9B). Although all model genes were markedly downregulated in tumor tissues, this does not necessarily imply a favorable patient prognosis as indicated by Figure 9C; Supplementary Figure S6. Notably, *CLDN4*, *VAV3*, *TEK*, *USP2*, *WNT9B*, and *CTSH* appear to correlate with the improved patient outcomes while *CHGA* and *GREM1* exhibit an inverse relationship, consistent with results shown in Supplementary Figure S1B. This finding suggests that not all genes significantly downregulated within tumor tissues are associated with positive prognosis for patients. Given that mRNA expression is pulsatile by nature, the expression of a gene is frequently regulated by multiple factors, and the entire process should be regarded as “dynamic” rather than “static”.

**FIGURE 10:**
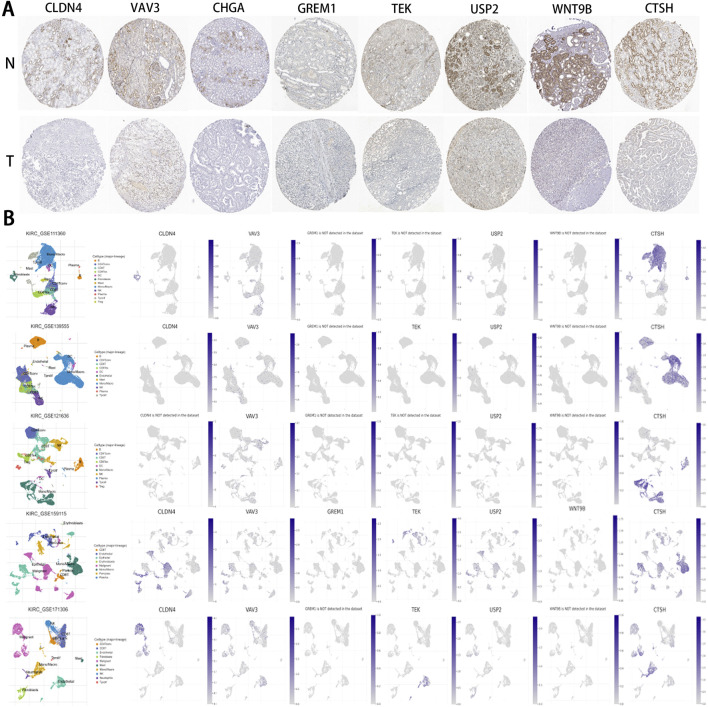
IHC and single-cell data results pertaining to IRPDGs. **(A)** Immunohistochemical (IHC) results for IRPDGs in ccRCC and normal tissues sourced from the HPA database. “N” denotes normal tissues, while “T” indicates tumor tissues. **(B)** Distribution of IRPDG expression across five single-cell datasets related to ccRCC from the TISCH database.

Based on the IHC results, the expression levels of the 8 IRPDGs were found to be lower in tumor tissues compared to normal tissues (Figure 10A), which is consistent with the findings presented in Figure 9A. To explore the recognition of IRPDGs by various immune cell subtypes, we selected multiple single-cell datasets from the TISCH database pertaining to ccRCC. As illustrated in Figure 10B, with the exception of missing data regarding *CHGA* within the database, the expression profiles of the remaining seven model genes appeared to correlate with different immune cell subtypes, indicating their potential for diverse immunological effects.

### 3.10 *In vitro* anti-cancer effect and preliminary mechanistic investigation of Zeb

**FIGURE 11:**
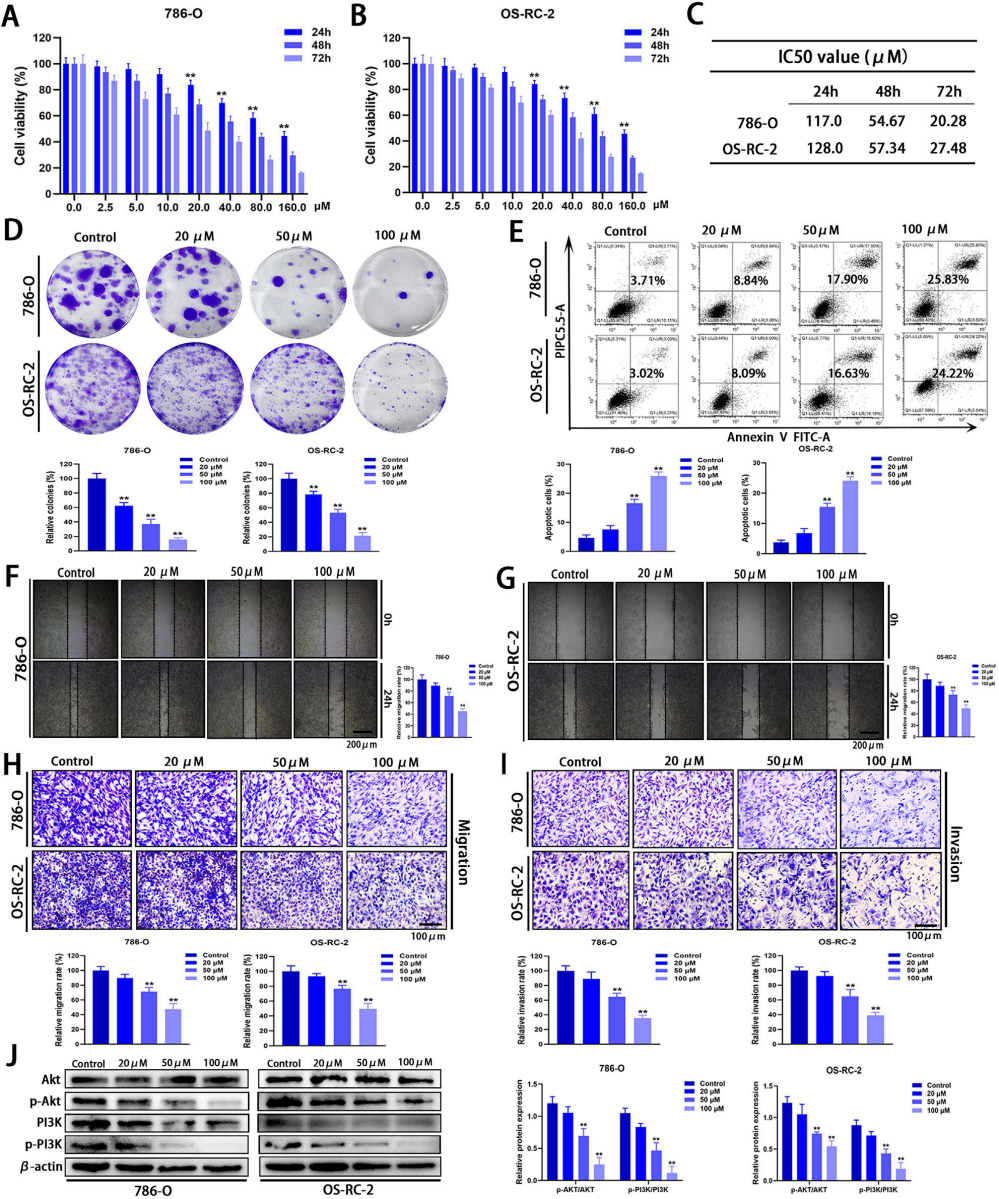
*In vitro* anti-cancer effects of Zeb. **(A, B)** MTT assays demonstrating that Zeb suppressed 786-O and OS-RC-2 cell viability time- and dose-dependently. **(C)** IC50 values of Zeb for 786-O and OS-RC-2 cell viability at 24, 48, and 72 h. **(D)** Colony formation assays for assessing the impact of Zeb on the proliferation of both cell lines. **(E)** Flow cytometry evaluating whether Zeb affected 786-O and OS-RC-2 cell apoptosis dose-dependently. **(F, G)** Scratch assays analyzing whether Zeb affected migration of both cell lines dose-dependently. **(H)** Transwell assays confirming the effects of Zeb on migration. **(I)** Transwell assays exploring the impact of Zeb on the invasion of both cell lines. **(J)** Western blot detecting the expression of Akt, p-Akt, PI3K and p-PI3K in cell lines. (**p < 0.01 *versus* Control; n = 3).

Considering that Zeb may influence signaling pathways associated with the progression of ccRCC, its anti-cancer activity *in vitro* was assessed using the MTT assay. Zeb exhibited significant inhibition against 786-O and OS-RC-2 cells at 24, 48 and 72 h in a dose- and time-dependent manner (P < 0.01; Figures 11A, B). The IC50 values are presented in Figure 11C. Treatment with 20 μM Zeb substantially decreased cell viability at 24 h (P < 0.01), therefore, Zeb at 20, 50 and 100 μM was chosen for later analyses. In the colony formation assay, the relative numbers of colonies formed by cells in different dose groups dramatically decreased relative to control group after 24 h of treatment with Zeb dose-dependently (P < 0.01; Figure 11D). Flow cytometry-based apoptosis assay revealed comparable results (P < 0.01; Figure 11E), indicating the dose-dependent pro-apoptotic effect of Zeb.

Tumor metastasis is a primary factor contributing to the elevated mortality rate and is closely associated with a fundamental cellular behavior known as cell motility (Li et al., 2016). Scratch and Transwell assays were performed to analyze how Zeb affected cell migration and invasion. The relative area of the intercellular gap on both sides of the Zeb-treated cells apparently decreased relative to control group (P < 0.01; Figures 11F, G), suggesting that Zeb effectively inhibited the migratory capacity of ccRCC cells. This finding was further validated by Transwell migration assays (P < 0.01; Figure 11H). Additionally, after treatment with Zeb for 24 h, the relative number of 786-O and OS-RC-2 cells invading lower chamber through extracellular matrix gel significantly decreased (P < 0.01, Figure 11I). These results indicate that Zeb efficiently suppressed both the migratory and invasive abilities of ccRCC cells dose-dependently.

To preliminarily investigate the molecular mechanisms by which Zeb influences the progression of ccRCC, we focused on the PI3K-Akt signaling pathway and validated our findings using Western blot analysis. As shown in Figure 11J, Zeb treatment significantly reduced the phosphorylation levels of PI3K and Akt in a dose-dependent manner (P < 0.01). These results demonstrate that Zeb has the potential to inhibit the PI3K-Akt signaling pathway through specific docking of core hub targets.

### 3.11 Inhibition of ccRCC solid tumor growth by Zeb and assessment of its toxicity

**FIGURE 12:**
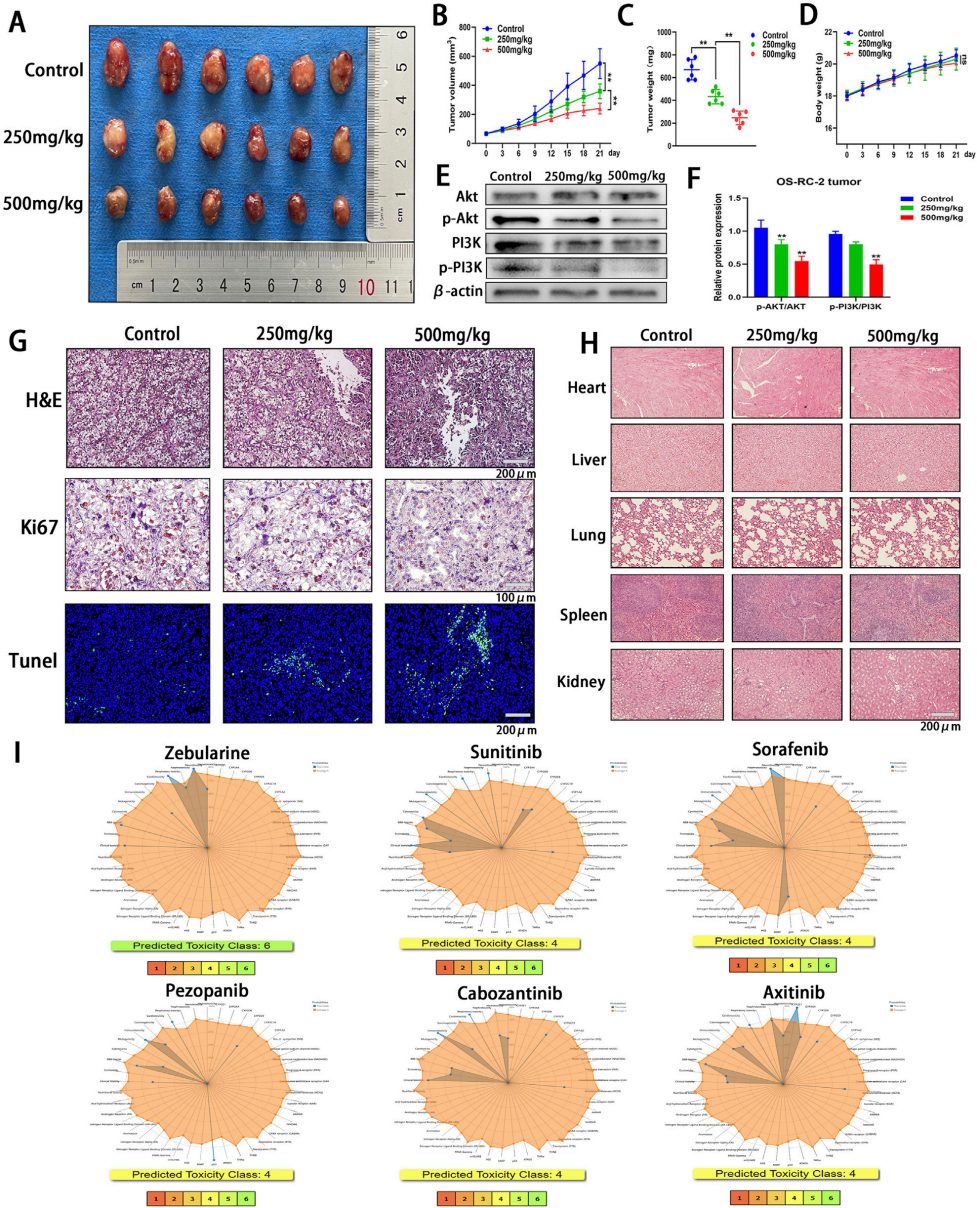
*In Vivo* Evaluation of the Antitumor Efficacy and Safety Profile of Zeb. **(A)** Tumor images from various treatment groups and the control group at the conclusion of the experiment. **(B)** Variations in the tumor growth curve throughout the entire Zeb treatment duration. **(C)** The distribution of tumor weights among each group at the conclusion of the experiment. **(D)** The body weight variation curves of the three groups of mice throughout the entire Zeb treatment period. **(E, F)** Western blot detecting the expression of Akt, p-Akt, PI3K and p-PI3K in tumor tissues. **(G)** Histological analyses, including H&E staining, Ki67 immunohistochemistry, and TUNEL fluorescence staining, were conducted on tumor sections from both the treatment and control groups. **(H)** Following the completion of Zeb treatment, histological sections of the primary organs from both the experimental and control groups of mice were prepared and subjected to H&E staining. **(I)** The ProTox database predicting the toxicity distribution of Zeb in relation to five major therapeutic drugs for the treatment of metastatic ccRCC. (**p < 0.01).

To assess the therapeutic efficacy of Zeb against ccRCC, an subcutaneous heterologous ccRCC model was established (Figure 12A). Zeb was administered orally at doses of 250 mg/kg or 500 mg/kg every other day. Tumor size was recorded every 3 days once the tumor volume reached 50 mm³. As illustrated in Figures 12A, B, treatment with 250 mg/kg of Zeb significantly inhibited the growth of ccRCC tumors, while the higher dose at 500 mg/kg demonstrated greater effectiveness compared to the relative lower dose (P < 0.01). Comparable results were observed for final tumor weight (P < 0.01; Figure 12C). Notably, throughout the observation period, the body weight of the mice exhibited a consistent increase (Figure 12D), indicating that Zeb has favorable biocompatibility. Additionally, Zeb intervention also suppressed the activation of the PI3K-Akt signaling pathway in tumor tissues in a dose-dependent manner (Figures 12E, F).

Subsequently, tumor tissues were sectioned for analysis. H&E staining revealed the presence of necrotic cancer cells in ccRCC tumors following Zeb treatment (Figure 12G). The anti-proliferative activity of Zeb was evaluated using Ki67 immunohistochemistry and apoptotic characteristics of the tumor tissue was assessed through TUNEL detection. As illustrated in Figure 12G, a negative correlation was observed between the number of Ki67-positive tumor cells and TUNEL staining results, indicating that high-dose Zeb possesses anti-ccRCC growth capacity.

Next, we sectioned the heart, liver, lung, spleen, and kidney tissues from mice and conducted H&E staining. The results indicated that no significant damage was observed in these major organs (Figure 12H), further corroborating the superior biocompatibility of Zeb. Additionally, we utilized the ProTox database to predict the biological toxicity of Zeb alongside key first-line drugs for metastatic ccRCC (Figure 12I). The findings revealed that Zeb may impair seven human functions, whereas Sunitinib, Sorafenib, Cabozantinib, and Axitinib were associated with impairment of eight human functions. Notably, Pezopanib exhibited less toxicity than Zeb by affecting six functions. Furthermore, while Zeb was classified as Toxicity Class 6, the other drugs were categorized as Class 4. These findings suggest that Zeb has enhanced biocompatibility compared to other targeted therapies.

## 4 Discussion

The treatment landscape for ccRCC has undergone a revolutionary transformation, and immunotherapy has emerged as one of the most significant breakthroughs (4, 5). Immunotherapy is demonstrated as the efficient treatment for advanced, recurrent, and metastatic cancer patients. However, the response rate to conventional ICIs monotherapies that target *PD-1/PD-L1* and *CTLA-4* remains low, and primary or secondary drug resistance might develop during treatment (Ballesteros et al., 2021). While some patients may benefit from novel ICIs monotherapies, such as *LAG-3* (13), Interleukin-1 (*IL-1*) (Kadomoto et al., 2021), Angiopoietin-2 (*Ang2*) (Bessho et al., 2015), T-cell immunoglobulin and mucin-domain containing-3 (*TIM-3*) (Kato et al., 2021), T-cell immunoreceptor with immunoglobulin and tyrosine-based inhibitory motif domain (*TIGIT*) (Noel et al., 2023), as well as pathways such as C-C motif chemokine ligand 2/C-C chemokine receptor type 2 (*CCL2/CCR2*) (Kadomoto et al., 2021), the overall response rates vary considerably among individuals. This variability may be attributed to the individual variability among patients and the inherent complexity of the immune system. Although combinations of ICIs with bispecific antibodies, oncolytic viruses, vaccines, and chimeric antigen receptor (CAR) T cell therapy have presented promising avenues for improving patient outcomes, ongoing research is warranted to expand and optimize immunotherapeutic strategies for ccRCC (Meng et al., 2024). Currently, there is no universally recognized biomarker for predicting responses to immunotherapy in ccRCC patients (Hua et al., 2022), in this regard, identifying a robust set of biomarkers is critical for developing novel therapeutic agents.

Precise prognosis prediction and formulation of personalized clinical treatment strategies constitute the cornerstone of precision medicine (Arnedos et al., 2014). This study comprehensively integrated two independent datasets from distinct platforms. Given the relatively small sample size of GSE29069, potential biases and statistical errors might arise, and this dataset was not utilized as an independent test set. Therefore, 70% of TCGA dataset was randomized as the training set, whereas the rest 30% was merged with GSE29069 to create the test set. By incorporating clinical data from the training set, eight genes that were remarkably related to OS of ccRCC patients were obtained. These genes were used to construct the 8-gene model, which demonstrated robust and stable prognostic performance, and was validated in the test set. This 8-gene model provided a creditable approach to predict ccRCC prognosis. The robust association between the 8-gene model and clinicopathological parameters, including T and M stages, further substantiated the relation between high RS and ccRCC metastasis as well as progression.

Of those eight genes detected, Claudin-4 (*CLDN4*) is a crucial structural protein located within tight junctions (TJ) of epithelial cells. *CLDN4* exerts an important effect on epithelial development and maintenance of polarity (Song et al., 2017). Furthermore, as an integral component of TJ, it regulates cell-to-cell adhesion, thereby contributing to cancer progression and potentially influencing tumor invasiveness and metastasis. Dysregulation of *CLDN4* has been recognized as a common characteristic across different cancer types, like lung, gastric, breast, ovarian, and colorectal cancers (Hashimoto et al., 2019). VAV guanine nucleotide exchange factor 3 (*VAV3*), a member of *VAV* gene family, can activate *Rho* family GTPases. VAV3 accelerates cell proliferation of breast cancer, gastric cancer, endometrial cancer, osteosarcoma, and acute lymphoblastic leukemia, and accelerates cell migration and invasion of breast cancer, pancreatic cancer, gastric cancer, and osteosarcoma (Miao et al., 2023). Conversely, it has decreased expression in renal cancer, which may promote the infiltration of anti-cancer immune cells, highlighting its specific role in different cancer types (Chang et al., 2024). Chromogranin A (*CHGA*), also known as parathyroid hormone-related protein 1 (*PTHrP1*), is a neuroendocrine secretory protein of the granule family and is localized within the secretory vesicles of neurons and endocrine cells (Helman et al., 1988). *GREMLIN1* (*GREM1*) belongs to the structurally and functionally related secretory cysteine knot protein family, and functions to isolate and inhibit the activity of multifunctional bone morphogenetic proteins (*BMPs*), thereby promoting the activation of cancer-associated fibroblasts and cancer stem cells (Gao et al., 2023). *CHGA* and *GREM1* are the only two high-risk genes within the model, suggesting that they are associated with poor prognosis of ccRCC. The tyrosine kinase receptor (*TEK*), initially characterized as a receptor specific to endothelial cells, functions in conjunction with its ligand, vascular endothelial growth factor (*VEGF*), as the co-regulator for vascular maturation and angiogenesis (Bessho et al., 2015; Teichert et al., 2017). Its depletion results in significant alterations in vascular development and remodeling. Besides, changes in *TEK* expression have been observed in tumor tissues including breast cancer, gastric cancer, thyroid cancer, and ccRCC (Ha et al., 2019). Ubiquitin-specific protease 2 (*USP2*) represents the cysteine protease of the USP family. It reverses the ubiquitin-mediated protein degradation and exerts an important effect on tumorigenesis by contributing to abnormal proliferation, migration, invasion, apoptosis, and drug resistance (Nadolny et al., 2021). WNT9B is closely associated with tumors. The abnormal expression and dysregulation of various *WNT* signaling pathways mediated by *WNT* ligands is important for the initiation and development of most human malignant tumors, like tumors affecting nervous, digestive, respiratory, urogenital, and musculoskeletal systems (Nusse et al., 1984). Dysregulation of these *WNT* pathways is undoubtedly related to ccRCC development. The last model gene, cathepsin H (CTSH), is a lysosomal cysteine protease with universal expression, which participates in specific cell processes and overall protein turnover, including cell apoptosis, hormone progenitor processing, and antigen presentation. Its high expression predicts good prognosis of tumors (Floyel et al., 2014). Overall, each IRPDG makes different contributions to ccRCC prognosis, ensuring that every IRPDG in the model maximizes its contribution.

The Von Hippel-Lindau (*VHL*) gene deletion or mutation is frequently a critical initial event for ccRCC occurrence (Jonasch et al., 2021). The 4 highly mutated genes associated with *VHL* include *PBRM1*, *SETD2*, *BAP1*, and *KDM5C* (Cotta et al., 2023). Their mutation frequency varies among studies, with *VHL* accounting for 49%–82%, *PBRM1* occupying 29%–41%, *SETD2* taking up 8%–30%, *BAP1* accounting for 6%–19%, and *KDM5C* holding 4%–15% of mutations (Mano et al., 2021; Kapur et al., 2013; Hakimi et al., 2013). It is traditionally believed that somatic mutations in *PBRM1*, *BAP1*, *SETD2*, and *KDM5C*, the genes known to influence chromatin remodeling and histone modification, lead to increased chromosomal instability and alterations in gene expression regulation that are associated with higher-grade tumors (Joosten et al., 2018). Notably, low-risk group in this study showed increased *VHL* and *PBRM1* mutation rates compared with high-risk group, demonstrating that higher rates of these mutations might be more favorable for the prognosis of ccRCC. Research has indicated that key genes implicated in ccRCC carcinogenesis (*VHL*, *PBRM1*, *BAP1*, and *SETD2*) are not strongly correlated with survival assessment (Petitprez et al., 2021). Hakimi et al. (Hakimi et al., 2013) demonstrated that *PBRM1* mutations did not significantly affect survival outcomes of ccRCC patients in both the MSKCC and TCGA cohorts. Similarly, *SETD2* mutations were lowly related to survival of ccRCC patients in TCGA cohort, while no significant association was observed in the MSKCC cohort. Additionally, activation of the hypoxia inducible factor (*HIF*) signaling pathway is contingent upon *VHL* mutations. However, certain VHL mutant ccRCC patients are resistant to inhibitors targeting the *HIF2α* signaling pathway, suggesting that VHL deficiency may influence the unidentified *HIF2α*-independent signaling pathways (Patel et al., 2022). Consequently, while *VHL* mutations are commonly observed in ccRCC, they do not necessarily dictate tumor progression or prognosis and may be associated with other intricate molecular mechanisms. Furthermore, after the silencing of all these 5 prevalent mutations in ccRCC-related genes (*VHL*, *PBRM1*, *BAP1*, *SETD2*, and *KDM5C*) from primary tumors in metastatic ccRCC patients who underwent palliative nephrectomy, various associations between mutation status and survival rates were observed. To be specific, *SETD2* and *KDM5C* mutations were correlated with improved OS, whereas *BAP1* mutations were linked to reduced OS, and *PBRM1* mutations showed no significant association with OS (Tennenbaum et al., 2017). Therefore, due to the inherent heterogeneity in the ccRCC genome, a comprehensive characterization of its drivers may be compromised. Thus far, tissue data remain essential for fully elucidating the genetic features of any specific tumor. As such, targeted therapies that aim to reduce the driving factors for the heterogeneity of the disease itself may be the future direction of treatment for ccRCC.

Thorsson et al. performed a cluster analysis of 30 non-hematological cancer types based on 160 immune-related feature scores, and identified 6 “immune subtypes” (C1-C6) (Vesteinn et al., 2018). The C4 and C6 subtypes were characterized by a predominance of M2 macrophages and the low lymphocyte infiltration rate, which were correlated with poor TME-related immune prognosis. In contrast, the C2 subtype exhibited the greatest M1/M2 macrophage polarization ratio and robust CD8 signaling. Additionally, the higher tumor proliferation rates contributed to an evolving IFN-γ-type immune response. C3 was defined as Th17 and Th1 gene upregulation with low-to-moderate tumor cell growth, and both subtypes were predictive of superior tumor prognoses. Based on our findings, high-risk group possessed greater proportions of C4 and C6 subtypes while a lower proportion of C3 than low-risk group, validating the accuracy of IRGPI. Notably, C2 subtype appeared to be less prevalent in low-risk group. Such discrepancy may arise from the fact that C2 tumors exhibit greater aggressiveness than C3 tumors in specific cases, accompanied by an ongoing IFN-γ immune response that is insufficient to control the rapid proliferation of C2 tumors. Additionally, it is possible that tumors classified as C2 have been reshaped by existing IFN-γ-type immune cell infiltration and have evaded the immune recognition. This phenomenon also elucidates why genes related to antigen processing and presentation are frequently lost in immuno-edited tumors, thereby impacting the prognosis of patients with C2-subtype tumors. Nevertheless, definitive conclusions should be validated with larger sample sizes.

Considering the intricate relationship between models and immunity, immune cell infiltration between the two subgroups were analyzed with CIBERSORT and ESTIMATE algorithms. As a result, the high-risk group showed increased immune cell infiltration levels, such as activated CD4 memory T cells, CD8 T cells, Tregs, follicular helper T cells, and M0 macrophages, particularly Tregs, suggesting a state of functional impairment in tumor-infiltrating T cells (Díaz-Montero et al., 2020). Conversely, the low-risk group displayed increased proportions of monocytes, activated CD4 memory T cells, resting dendritic cells, M1 macrophages, and resting mast cells, indicating that the TME changed to a more tumor-suppressive phenotype. ccRCC mediates immune dysfunction through inducing Tregs while simultaneously up-regulating checkpoints to inhibit effector T cell activity and antigen-presenting cell function (Díaz-Montero et al., 2020). Furthermore, the high-risk group demonstrated remarkably higher immune/estimate scores, reflecting the greater complexity within TME. In this study, the TIDE score was used as a predictive indicator for response to checkpoint blockade therapy and the associated survival benefits (Qin et al., 2023). The elevated level of T cell dysfunction and TIDE score observed in the high-risk group indicated that TME was characterized by a pronounced state of immune evasion. This phenomenon elucidates why alterations and remodeling of the TME are responsive to the increasing immune cell infiltration, ultimately facilitating immune escape, even though the high-risk group is classified into the more favorable C2 immune subtype (Joosten et al., 2018). Additionally, MSI serves as a marker of genetic instability and is increasingly adopted for identifying patients probably benefiting from targeted therapies, immunotherapy, and advanced systemic treatments (Qin et al., 2023; Joshi and Badgwell, 2021). Our findings indicated that the low-risk group exhibited an elevated MSI score, suggesting that ICIs could confer advantages for this cohort, consistent with the observed lower level of dysfunction and TIDE score indicative of the diminished potential for immune evasion. Nonetheless, it is important to note that the prognostic performance of our model for ICIs treatment remains limited, and further analysis is warranted to clarify its precise functionalities and broader applicability.

Additionally, difference in the sensitivity to first-line therapies (Huang et al., 2019; Roulin et al., 2011) for metastatic ccRCC between the two subgroups were analyzed using the “pRRophetic” package. As a result, high-risk group showed higher sensitivity to Sunitinib than to Sorafenib and Pazopanib. This discrepancy may be attributed to the distinct mechanisms of action associated with different targeted agents. Notably, the hypoglycemic agent Metformin also demonstrated enhanced sensitivity in the low-risk group, and there were more pronounced difference in sensitivity between the two subgroups than those observed for Sorafenib and Pazopanib. These results offer a novel framework for future clinical studies that incorporate low-risk patients, particularly those with both ccRCC and type 2 diabetes.

Finally, Zeb was identified as the drug with the lowest negative score based on the differential expression profiles between the two subgroups. Zeb is a DNA methyltransferase inhibitor that induces cell cycle arrest and apoptosis through intrinsic apoptotic pathway through activating BAX and BAK (Ruiz-Magana et al., 2012). It has been demonstrated with anti-cancer effects in various malignancies, including gastric cancer, breast cancer, glioma, and leukemia (Ruiz-Magana et al., 2012; Chen et al., 2012; Lai et al., 2021). To elucidate the mechanisms by which Zeb influences the progression of ccRCC, we employed network pharmacology and molecular docking approaches to investigate a range of potential molecular pathways. Additionally, we experimentally validated Zeb’s anticancer activity and its primary targeting of the PI3K-Akt signaling pathway. Nevertheless, the anti-cancer activity of Zeb against ccRCC was suboptimal, with IC50 values for 786-O cells being 117, 54.67, and 20.28 μM after 24, 48, and 72 h, respectively. Similarly, high IC50 values were observed for OS-RC-2 cells at 128, 57.34, and 27.48 μM, which were significantly higher than those of Sunitinib, Sorafenib, and Pazopanib (Huang et al., 2019; Roulin et al., 2011). Furthermore, *in vivo* studies indicated that achieving satisfactory anti-tumor effects required substantially higher doses of Zeb compared with the commonly accepted dosages of Sunitinib and Sorafenib. This discrepancy may be attributed to the fact that subgroups in our model were delineated based on OS data from TCGA patients, which were independent of variations among cell lines, and the model features were constructed from a comprehensive perspective. In contrast, Zeb specifically targeted the high-risk IRGPI populations, whereas Sunitinib, Sorafenib, and Pazopanib were directed towards the general ccRCC population. Extensive research is required to elucidate whether Zeb exerts a significant influence on the progression of high-risk ccRCC via specific mechanisms.

## 5 Conclusion

In summary, the IRGPI developed for ccRCC in this study demonstrates superior prognostic performance for ccRCC, and is externally validated in an independent cohort. Beyond its prognostic performance, IRGPI also provides insights into the molecular characteristics and tumor immunity. Additionally, a small molecule compound that target high-risk ccRCC is identified, its potential mechanisms of action are investigated through network pharmacology and molecular docking, and its antitumor effect and preliminary molecular mechanism are confirmed *in vitro* and *in vivo*. Our future research will concentrate on evaluating the prognostic efficacy of IRGPI in prospective cohorts and conducting clinical studies involving Zeb.

## Data Availability

The original contributions presented in the study are included in the article/Supplementary Material, further inquiries can be directed to the corresponding authors.
