# Supplementary material for: Zebularine showed anti-tumor efficacy in clear cell renal cell carcinoma
Source: Front Pharmacol. 2025 Feb 14;16:1531056. doi: 10.3389/fphar.2025.1531056 (PMC11868290; doi:10.3389/fphar.2025.1531056)
Supplement: Supplementary file 1 [file DataSheet4.docx]

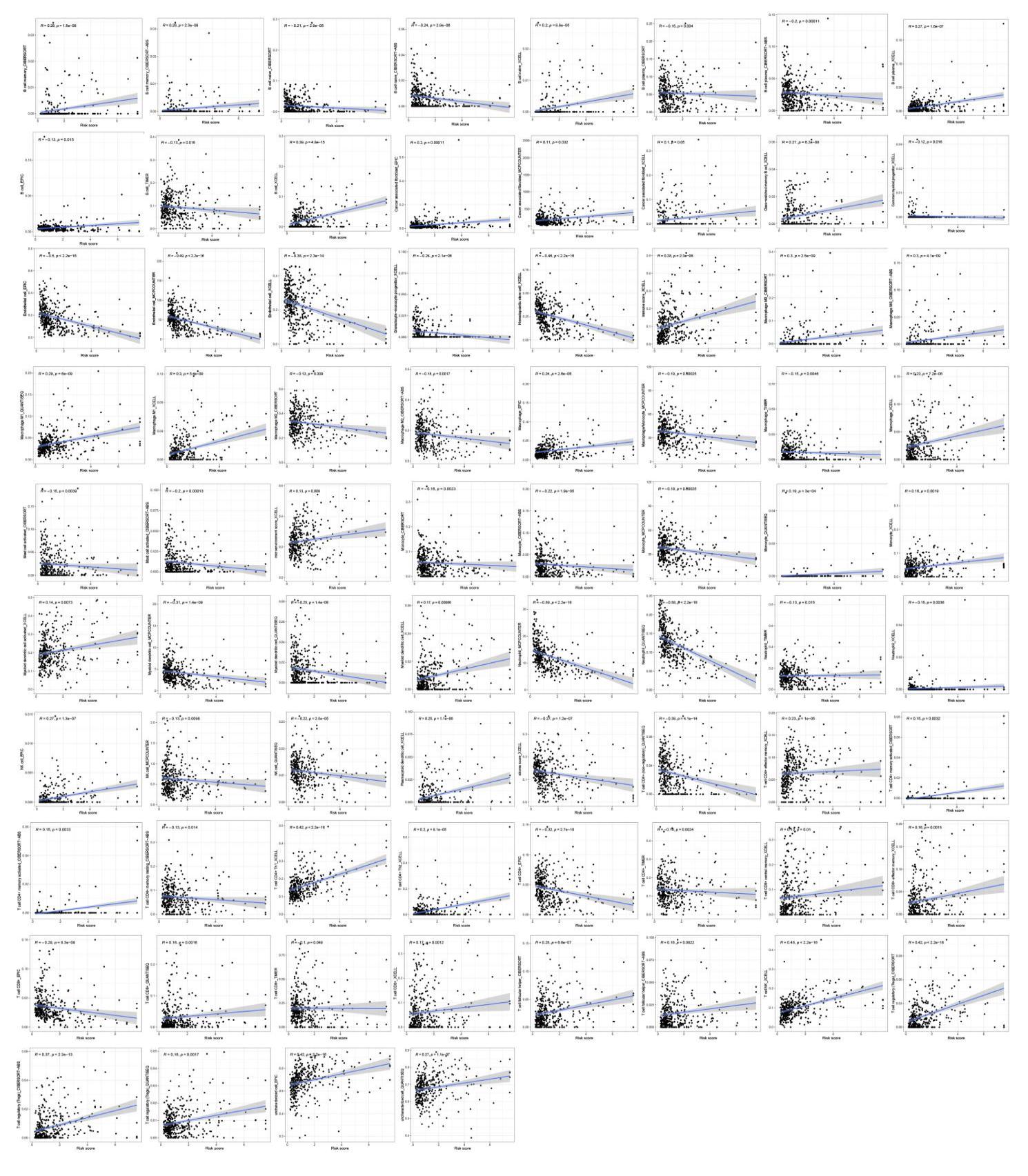


**SUPPLEMENTARY FIGURE S4:** Correlation plots illustrating the relationship between various immune cell types and RS, derived from seven algorithms including CIBERSORT, CIBERSORT-ABS, EPIC, MCPCOUNTER, QUANTISEQ, TIMER, and XCELL.
