# Supplementary figures and images for: Zebularine showed anti-tumor efficacy in clear cell renal cell carcinoma
Source: Front Pharmacol. 2025 Feb 14;16:1531056. doi: 10.3389/fphar.2025.1531056 (PMC11868290; doi:10.3389/fphar.2025.1531056)

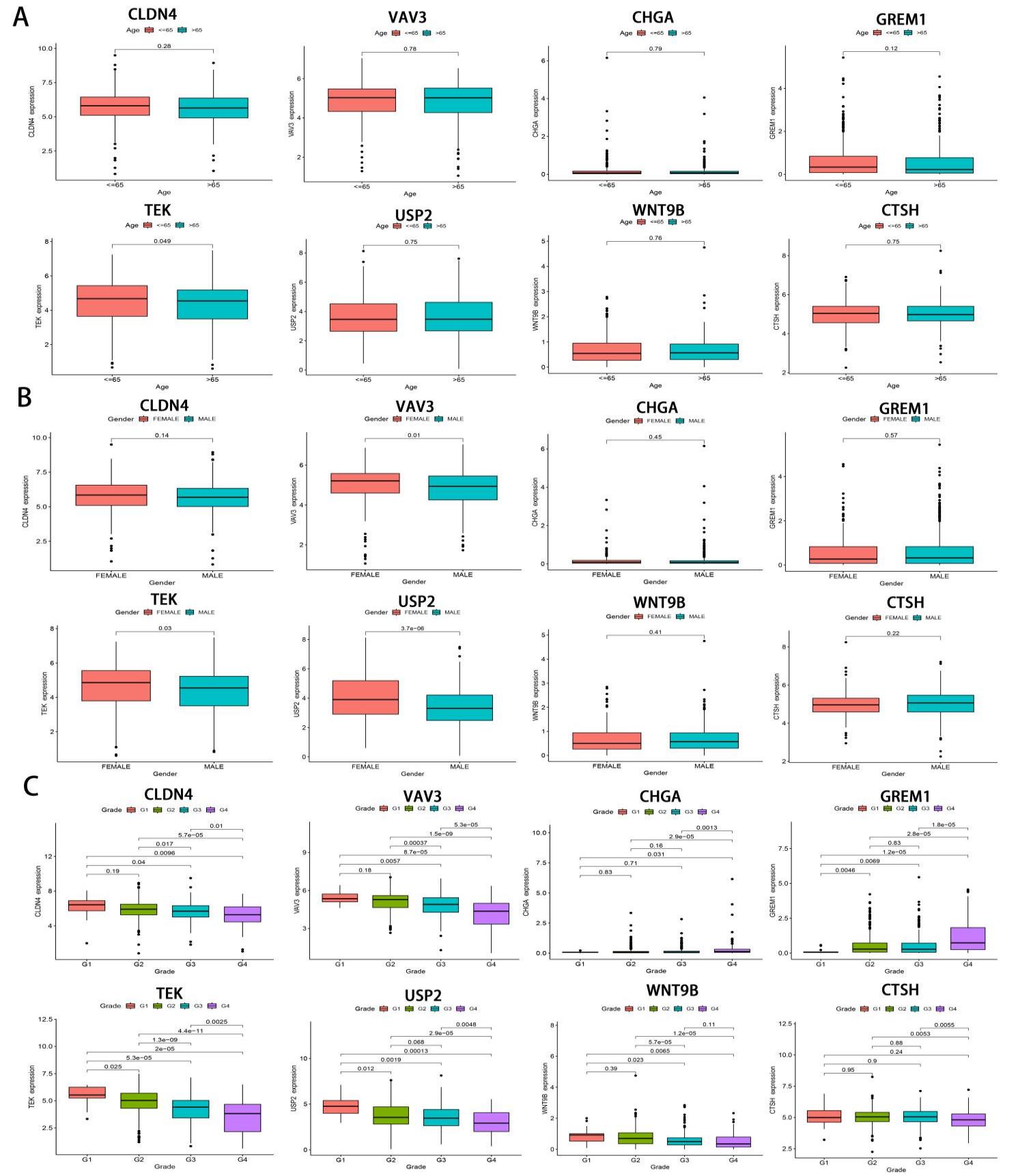


**SUPPLEMENTARY FIGURE S6:** The association between 8 IRPDGs and **(A)** age, **(B)** gender, and **(C)** grade.

Supplement: Supplementary file 2 [file DataSheet6.docx]
