# Supplementary material for: Zebularine showed anti-tumor efficacy in clear cell renal cell carcinoma
Source: Front Pharmacol. 2025 Feb 14;16:1531056. doi: 10.3389/fphar.2025.1531056 (PMC11868290; doi:10.3389/fphar.2025.1531056)
Supplement: Supplementary file 3 [file Table1.docx]

**SUPPLEMENTARY TABLE :** RT-qPCR primer sequences for eight IRPDGs

| Gene | Orientation | Primer sequence (5’ to 3’) |
| --- | --- | --- |
| CLDN4 | Forward | ACTGCCTGGAGGATGAAAGC |
|  | Reverse | GACACCGGCACTATCACCAT |
| VAV3 | Forward | TGCAAGCAGAGACCGAACTTATT |
|  | Reverse | CTGAACCCTTCCTTGAGAGAATGA |
| CHGA | Forward | CACTCCGAGGAGATGAACGG |
|  | Reverse | CTGGCTGCTCTGGTTCTCAA |
| GREM1 | Forward | CCCAGGAAGTCCCAGACCTA |
|  | Reverse | TCCACACTCATGCACACGAA |
| TEK | Forward | CATCCTTGGCTCTGCTGGAA |
|  | Reverse | TTTGGAAGGCTTGGGCCATT |
| USP2 | Forward | GAGTTCAAGACCCAGATCCAGAG |
|  | Reverse | GTCACTCGGTTCACCTCGTTATG |
| WNT9B | Forward | GTGGGCATCAAGGCTGTGAA |
|  | Reverse | CCGAGTCATAGCGCAGTTTC |
| CTSH | Forward | CTGTGAAAAATCAGGGTGCC |
|  | Reverse | GAAGACAACTGAGGCTGCAA |
| GAPDH | Forward | GCACCGTCAAGGCTGAGAAC |
|  | Reverse | TGGTGAAGACGCCAGTGGA |
