# Supplementary material for: Zebularine showed anti-tumor efficacy in clear cell renal cell carcinoma
Source: Front Pharmacol. 2025 Feb 14;16:1531056. doi: 10.3389/fphar.2025.1531056 (PMC11868290; doi:10.3389/fphar.2025.1531056)
Supplement: Supplementary file 4 [file DataSheet3.docx]

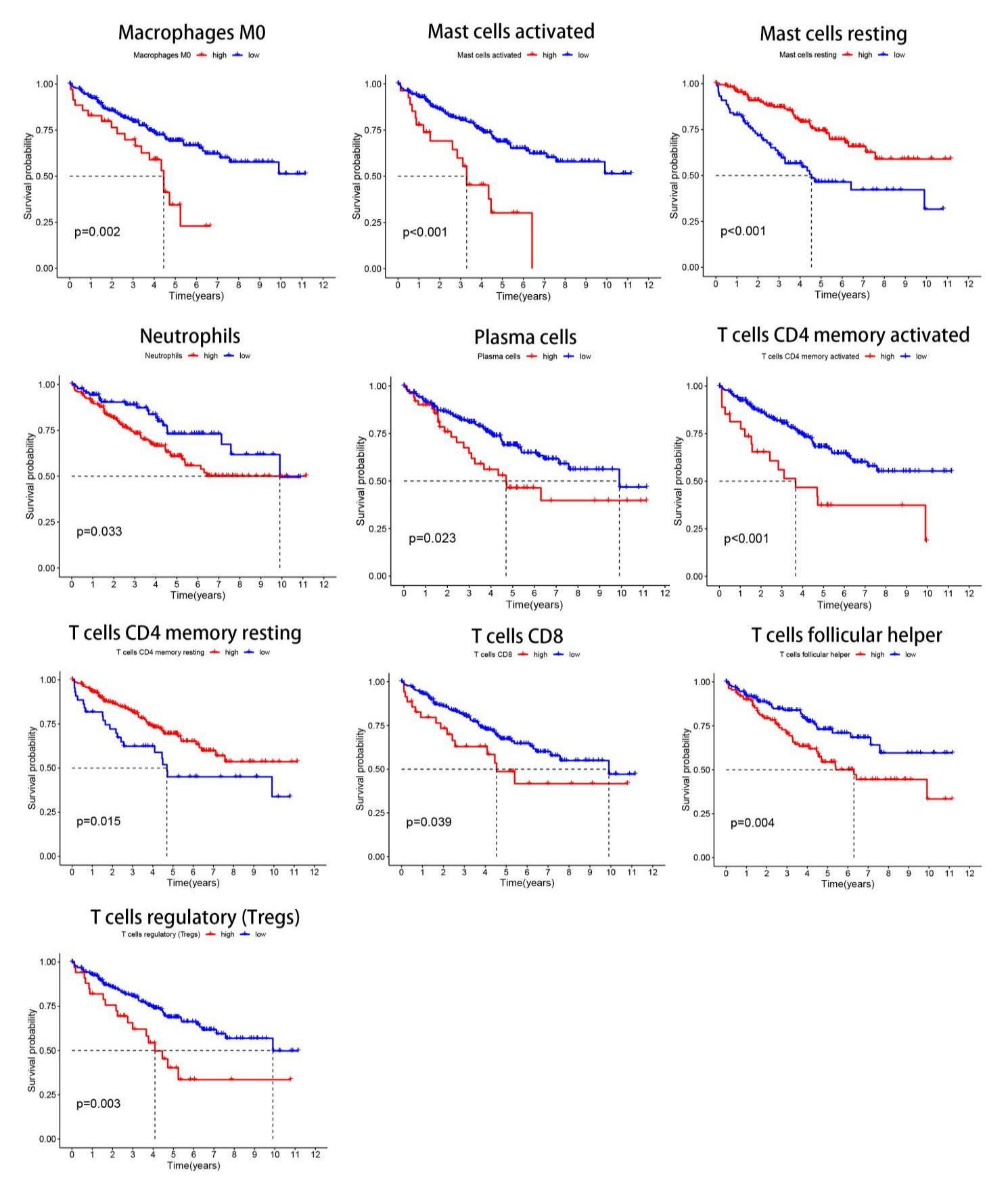


**SUPPLEMENTARY FIGURE S3:** Differential analysis of survival curves for ten immune cell types between high- and low-risk subgroups.
