# Supplementary material for: Zebularine showed anti-tumor efficacy in clear cell renal cell carcinoma
Source: Front Pharmacol. 2025 Feb 14;16:1531056. doi: 10.3389/fphar.2025.1531056 (PMC11868290; doi:10.3389/fphar.2025.1531056)
Supplement: Supplementary file 5 [file DataSheet5.docx]

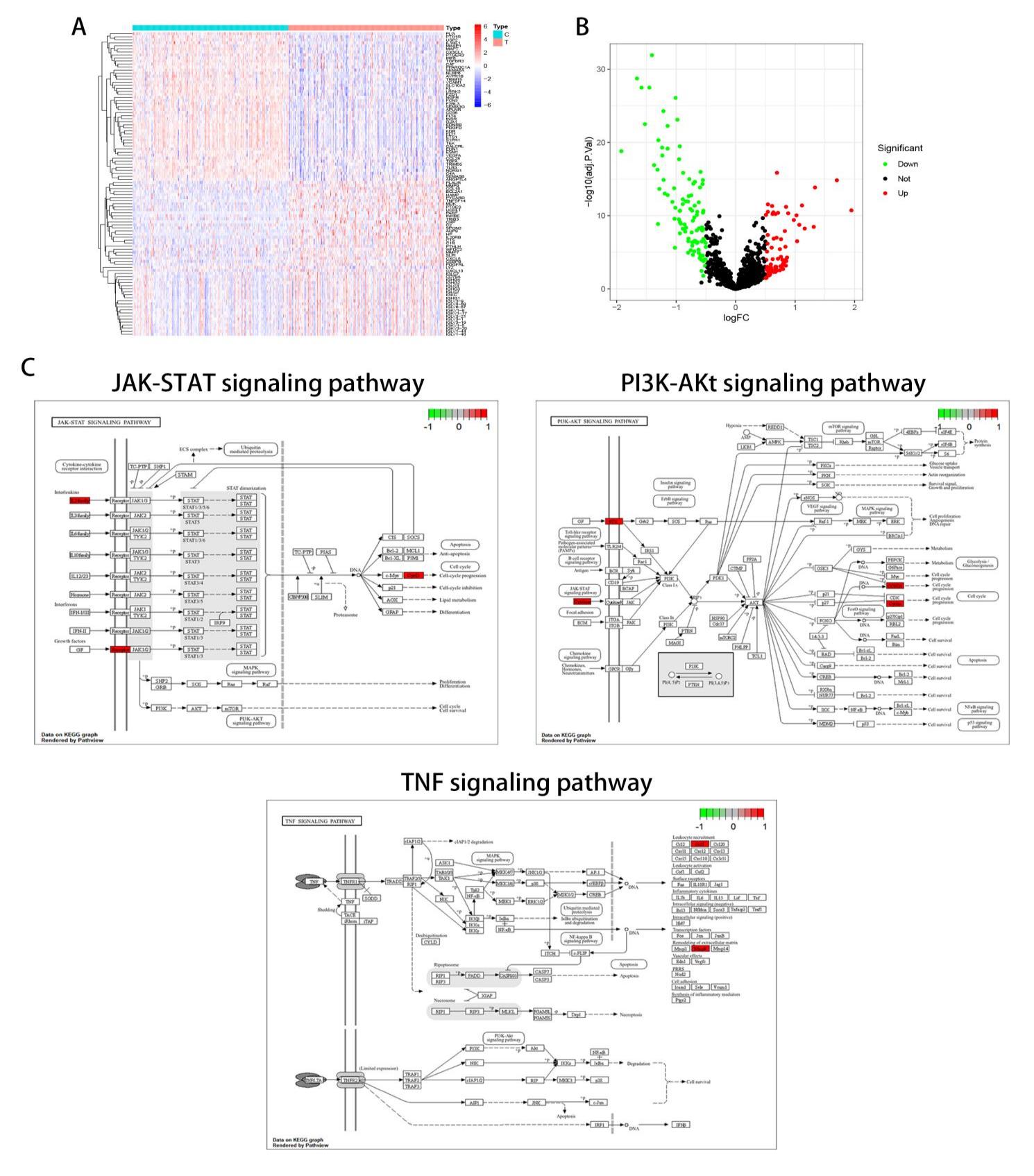


**SUPPLEMENTARY FIGURE S5: (A)** Heatmap depicting mIRDEGs between high- and low-risk subgroups. **(B)** Volcano plot illustrating mIRDEGs between high- and low-risk subgroups. **(C)** Corresponding schematic representations of JAK−STAT, PI3K−Akt, and TNF pathways.
