# Supplementary material for: Zebularine showed anti-tumor efficacy in clear cell renal cell carcinoma
Source: Front Pharmacol. 2025 Feb 14;16:1531056. doi: 10.3389/fphar.2025.1531056 (PMC11868290; doi:10.3389/fphar.2025.1531056)
Supplement: Supplementary file 7 [file DataSheet1.docx]

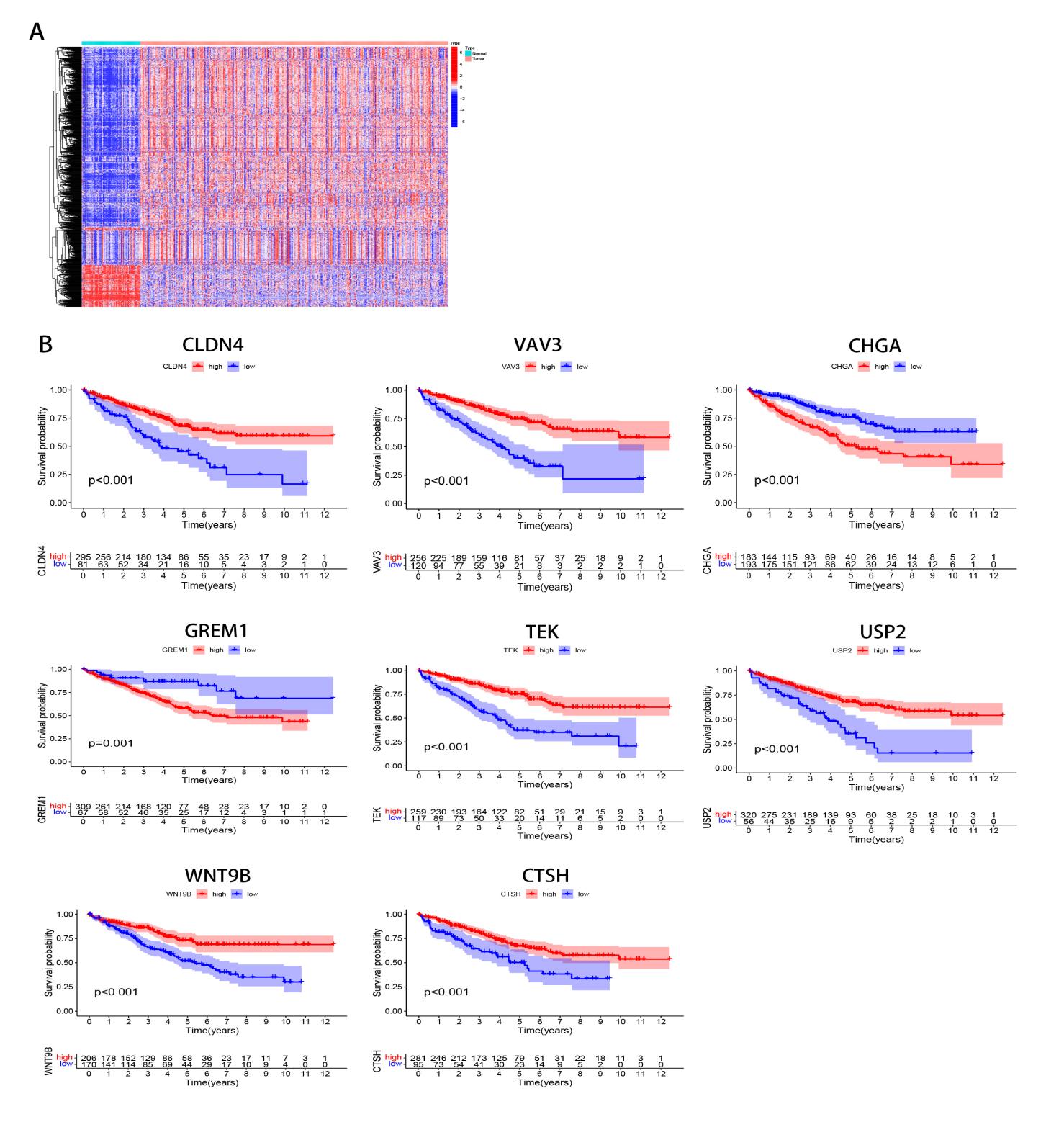


**SUPPLEMENTARY FIGURE S1:** **(A)** Heatmap illustrating the IRDEGs between ccRCC tumor samples and corresponding normal tissue samples. **(B)** Differential analysis of survival curves for each IRPDG.
